# Flagellum-driven cargoes: Influence of cargo size and the flagellum-cargo attachment geometry

**DOI:** 10.1371/journal.pone.0279940

**Published:** 2023-03-10

**Authors:** Albert J. Bae, Raheel Ahmad, Eberhard Bodenschatz, Alain Pumir, Azam Gholami

**Affiliations:** 1 Lewis & Clark College, Portland, Oregon, United States of America; 2 Max Planck Institute for Dynamics and Self-Organization, Göttingen, Germany; 3 Center for Cancer Research and Center for Engineering in Medicine and Surgery, Harvard Medical School and Massachusetts General Hospital, Charlestown, Massachusetts, United States of America; 4 Institute for Dynamics of Complex Systems, University of Göttingen, Göttingen, Germany; 5 Laboratory of Atomic and Solid-State Physics and Sibley School of Mechanical and Aerospace Engineering, Cornell University, Ithaca, New York, United States of America; 6 Laboratoire de Physique, Ecole Normale Supérieure de Lyon, Université Lyon 1 and CNRS, Lyon, France; 7 New York University Abu Dhabi, Abu Dhabi, United Arab Emirates; Coastal Carolina University, UNITED STATES

## Abstract

The beating of cilia and flagella, which relies on an efficient conversion of energy from ATP-hydrolysis into mechanical work, offers a promising way to propel synthetic cargoes. Recent experimental realizations of such micro-swimmers, in which micron-sized beads are propelled by isolated and demembranated flagella from the green algae *Chlamydomonas reinhardtii* (*C. reinhardtii*), revealed a variety of propulsion modes, depending in particular on the calcium concentration. Here, we investigate theoretically and numerically the propulsion of a bead as a function of the flagellar waveform and the attachment geometries between the bead and the flagellum. To this end, we take advantage of the low Reynolds number of the fluid flows generated by the micro-swimmer, which allows us to neglect fluid inertia. By describing the flagellar waveform as a superposition of a static component and a propagating wave, and using resistive-force theory, we show that the asymmetric sideways attachment of the flagellum to the bead makes a contribution to the rotational velocity of the micro-swimmer that is comparable to the contribution caused by the static component of the flagellar waveform. Remarkably, our analysis reveals the existence of a counter-intuitive propulsion regime in which an increase in the size of the cargo, and hence its drag, leads to an increase in some components of the velocity of the bead. Finally, we discuss the relevance of the uncovered mechanisms for the fabrication of synthetic, bio-actuated medical micro-robots for targeted drug delivery.

## 1 Introduction

Cilia and flagella are fundamental units of motion in various biological systems. These hair-like cellular protrusions share a common conserved 9+2 microtubule-based structure, and beat to accomplish a variety of biological tasks. Examples are surface fluid flows generated by ciliary carpet in the human respiratory tract to remove pollutants [1], cilia-driven cerebrospinal fluid transport in the mammalian brain to deliver nutrients and important signaling molecules [2], ciliary flow in the Fallopian tube to assist sperm transport to the fertilization site [3], and propulsion of green algae *C. reinhardtii* that swims by breaststroke-like motion of its two flagella [4–6].

**Fig 1:**
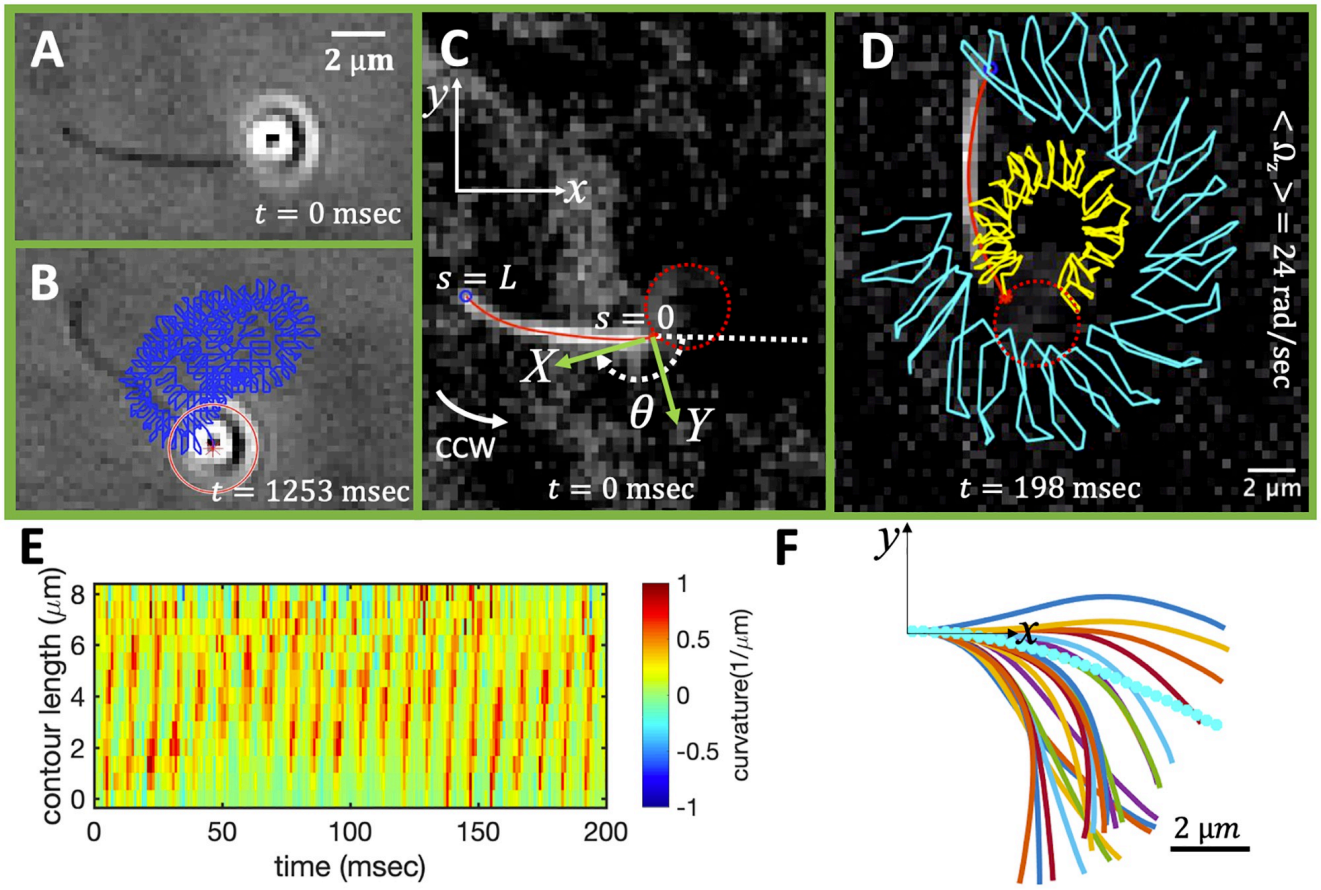
An exemplary experiment showing a flagellum-based bead propulsion. A) An isolated and demembranated flagellum (known as axoneme) from green algae *C. reinhardtii* is attached to a 1 micron-sized bead. The axoneme is reactivated with 1 mM ATP and beats at around 110 Hz (see S1 Video). B) Over time, the position of the center of the bead, its evolution represented by the blue curve, is propelled on a helical-like trajectory (see S2 Video). (C) The definition of the laboratory and the swimmer-fixed frame. As the flagellum beats, the micro-swimmer swims counterclockwise (CCW) in the microscope’s field of view effectively in 2D. D) The traces of the basal (yellow line) and distal tip (cyan line) of the flagellum tracked for 198 sec. (E) Curvature waves initiate at the basal end of axoneme (*s* = 0) which is attached to the bead, and propagate toward the distal tip (*s* = *L*) at the frequency of about 110 Hz. F) The axonemal shapes averaged over one beat cycle results in a circular arc with mean curvature of about -0.2 *μ*m^−1^. This static component of the axonemal curvature results in a curved swimming trajectory and in the absence of this component, the bead is propelled on a straight trajectory. The experimental techniques used to record the motion of the beads and the flagella are described in Ref. [14]. Please note that in this experiment, the bead-axoneme attachment appears to be asymmetric but 3D microscopy techniques are required to distinguish a symmetric versus an asymmetric attachment.

In recent years, there has been a great interest in the field of targeted drug delivery and assisted fertilization to integrate cilia and flagella as efficient energy conversion modules into bio-compatible micro-swimmers. Autonomous flagella-driven motility of various biological species, mainly *E. coli* and sperm, are utilized as bio-actuators to provide an efficient cargo transport [7–13]. More recently, in the experiments by Ahmad et al. [14], axonemally-driven cargoes are fabricated by integration of isolated and demembranated flagella from *C. reinhardtii* (known as axonemes) with micron-sized beads (see Fig 1 and S1–S2 Videos). These ATP-reactivated axonemes, with a length of approximately 10 *μ*m, beat with an ATP-dependent frequency [15–17] and have an asymmetric waveform that can best be described as a base-to-tip traveling wave component superimposed on a circular arc with mean curvature of about -0.2 *μ*m^−1^. The static component of the axonemal curvature leads to a curved swimming trajectory of the micro-swimmer (see Fig 1D) [18–21]. In comparison, when the static curvature is strongly reduced, a micro-swimmer swims along an essentially straight path [14, 22–24]. Importantly, the static curvature of axonemes is highly dependent on the calcium concentration. Namely, increasing the calcium concentration beyond 0.05 mM reduces the static curvature of axonemes by one order of magnitude [14, 23, 24], thereby inducing a transition from circular to straight swimming trajectories of axonemally-propelled beads. In addition to the flagellar waveform, the axoneme-bead attachment geometry also plays a critical role in the cargo propulsion speed. As emphasized in Ref. [14], axonemes can attach to the bead symmetrically, with their tangent vector at the contact point passing through the bead center, or asymmetrically. Due to the limitations of 2D microscopy in Ref. [14], it was not experimentally possible to distinguish between these two types of bead-axoneme attachment and 3D microscopy techniques [25] are required to quantify the effect of attachment geometry on the propulsion speed. This symmetric versus asymmetric attachment has consequences on cargo propulsion dynamics and investigating this effect theoretically and numerically is the main focus of the present work.

Here, we investigate the effect of (i) various flagellar wave components, (ii) the size of the cargo (the bead), and (iii) the symmetric versus asymmetric flagellum-bead attachment on the swimming dynamics of a bead that is propelled by a model flagellum. We restrict ourselves to two-dimensional motion, which captures most of the experimental results in Ref. [14]. We use an approximate description of the flagellum waveform as a combination of a static curvature and a traveling wave component, and use resistive-force theory (RFT) [26, 27] to obtain analytical expressions for the translational and rotational velocities of a flagellum-propelled bead in the limit of small amplitude of curvature waves. We compare the resulting expressions to the results of simulations of the swimming trajectories. Our analysis reveals a surprising non-monotonic behavior of the mean translational and rotational velocities of the axonemally-driven bead as a function of the bead radius. Finally, our analysis shows that for a freely swimming axoneme, which rotates predominantly with its static component of the axonemal waveform, sideways bead attachment is sufficient to generate mean rotational velocities comparable to the rotation rates induced by the static curvature. This paper is structured as follows: first we briefly describe RFT, which we use to calculate the propulsion speed of the micro-swimmer as a function of the cargo size. In this approach, the specific details of the bead-axoneme attachment geometry are taken into account in the drag matrices of the bead and the axoneme. Next, we present our analytical approximations and numerical simulations to show the effect of the cargo size and of the symmetric versus asymmetric bead-axoneme attachment.

## 2 Materials and methods

### 2.1 RFT and calculations of mean translational and rotational velocities

The fluid flow generated by the swimming of small objects is characterized by very small Reynolds numbers. In this regime, viscous forces dominate over inertia and non-reciprocal motion is necessary to break the time-symmetry and generate propulsion (scallop theorem) [28, 29]. The micro-swimmer in our system consists of an axoneme (a filament of characteristic length *L* ∼ 10 *μ*m and radius 0.1 *μ*m), which is attached at one end to a micron-sized bead and swims in an aqueous solution of viscosity *μ* = 10^−3^ Pa s and density *ρ* = 10^3^ kg m^−3^. Given the characteristic axonemal wave velocity *V* = λ/*T* ≈ 0.5 mm s^−1^ (calculated for a typical axonemal beat frequency of 50 Hz and a wavelength which is comparable to the axonemal contour length *L*), the Reynolds number *Re* = *ρLV*/*μ* is small, no larger than ∼ 0.005. In this physical regime, Newton’s laws then consist of an instantaneous balance between external and fluid forces and torques exerted on the swimmer, i.e. **F**_ext_ + **F**_fluid_ = 0 and ***τ***_ext_ + ***τ***_fluid_ = 0. The force **F**_fluid_ and torque ***τ***_fluid_ exerted by the fluid on the axoneme-bead swimmer can be written as:

$$F_{\text{fluid}}=F_{\text{Bead}}+\int_{0}^{L}ds\;F_{\text{Axoneme}}\left(s,t\right),$$ (1)

$$\tau_{\text{fluid}}=\tau_{\text{Bead}}+\int_{0}^{L}ds\;r\left(s,t\right)\times F_{\text{Axoneme}}\left(s,t\right),$$ (2)

where **F**_bead_ and ***τ***_Bead_ are the hydrodynamic drag force and torque acting on the bead, and the integrals over the contour length *L* of the axoneme calculate the total hydrodynamic force and torque exerted by the fluid on the axoneme. The bead is propelled by the oscillatory shape deformations of the ATP-reactivated axoneme. At any given time, we consider axoneme-bead swimmer as a solid body with translational and rotational velocities **U**(*t*) and **Ω**(*t*) to be determined as explained below. **F**_fluid_ and ***τ***_fluid_ can be separated into propulsive part due to the relative shape deformations of the axoneme in the body-fixed frame and the drag part [30]:

$$\begin{array}{c} (\begin{array}{c} F_{\text{fluid}}\\ \tau_{\text{fluid}} \end{array})=(\begin{array}{c} F^{\text{prop}}\\ \tau^{\text{prop}} \end{array})-D(\begin{array}{c} U\\ \Omega \end{array})=(\begin{array}{c} F^{\text{prop}}\\ \tau^{\text{prop}} \end{array})-\left(D_{A}+D_{B}\right)(\begin{array}{c} U\\ \Omega \end{array}), \end{array}$$ (3)

where the 6 × 6 geometry-dependent drag matrix **D** is symmetric and non-singular (invertible) and is composed of drag matrix of the axoneme **D**_*A*_ and drag matrix of the bead **D**_*B*_. For a freely swimming axoneme-bead, which experiences no external forces and torques, **F**_fluid_ and ***τ***_fluid_ must vanish. As explained in the introduction, we restrict ourselves to 2-dimensional motion, which describes most of the experimental work described by Ref. [14]. In 2-dimensions, **D** is reduced to a 3 × 3 matrix and Eq 3 can be reformulated as:

$$\left(\begin{array}{c} U_{x}\\ U_{y}\\ \Omega_{z} \end{array}\right)={\left(D_{A}+D_{B}\right)}^{-1}(\begin{array}{c} F_{x}^{\text{prop}}\\ F_{y}^{\text{prop}}\\ \tau_{z}^{\text{prop}} \end{array}),$$ (4)

which we use to calculate translational and rotational velocities of the swimmer after determining the drag matrices **D**_*A*_ and **D**_*B*_, and the propulsive forces and torque $\left(F_{x}^{\text{prop}},F_{y}^{\text{prop}},\tau_{z}^{\text{prop}}\right)$.

We calculate $F_{x}^{\text{prop}}$, $F_{y}^{\text{prop}}$ and $\tau_{z}^{\text{prop}}$ in the body-fixed frame by selecting the basal end of the axoneme (bead-axoneme contact point) as the origin of the swimmer-fixed frame. As shown in Figs 1C and 3A, we define the local tangent vector at contour length *s* = 0 as **X**-direction, the local normal vector ***n*** as the **Y**-direction, and assume that ***z*** and **Z** are parallel. Here (***x***, ***y***, ***z***) denote an orthogonal lab-frame basis. We define *θ*_0_(*t*) = *θ*(*s* = 0, *t*) as the angle between ***x*** and **X** which gives the velocity of the bead in the laboratory frame as $U_{x}^{\text{Bead-Lab}}=\text{cos}\theta_{0}\left(t\right)U_{x}+\text{sin}\theta_{0}\left(t\right)U_{y}$ and $U_{y}^{\text{Bead-Lab}}=-\text{sin}\theta_{0}\left(t\right)U_{x}+\text{cos}\theta_{0}\left(t\right)U_{y}$. Furthermore, note that the instantaneous velocity of the axoneme in the lab frame is given by **u** = **U** + **Ω** × **r**(*s*, *t*) + **u**′, where **u**′ is the deformation velocity of the flagellum in the body-fixed frame, **U** = (*U*_*x*_, *U*_*y*_, 0) and **Ω** = (0, 0, Ω_*z*_) with Ω_*z*_ = *dθ*_0_(*t*)/*dt*.

**Fig 2:**
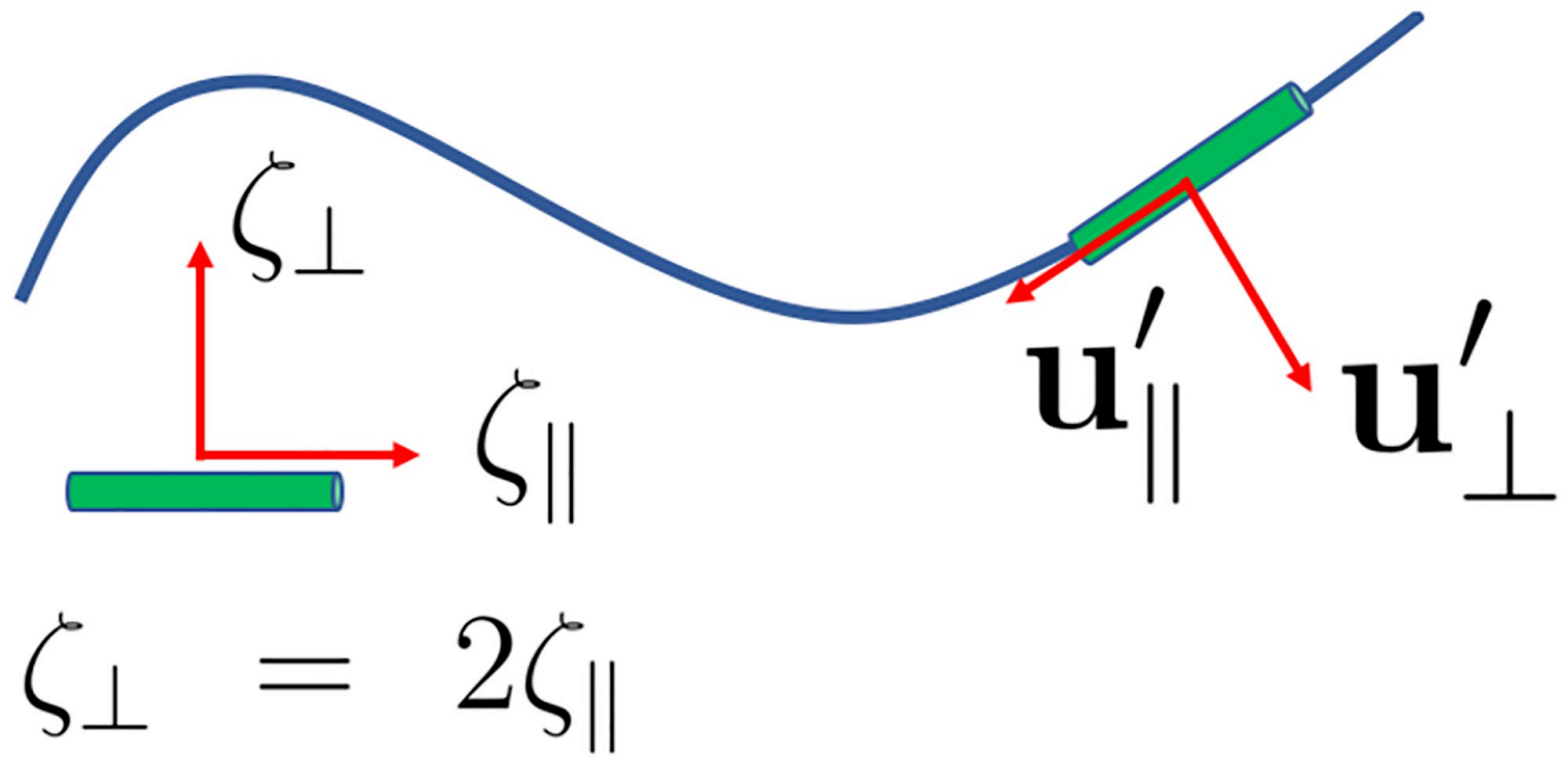
A schematic representation of the Resistance Force Theory (RFT) calculation. A flagellum, depicted by the blue line, is decomposed into small cylindrical segments moving at a velocity **u**′, which is decomposed as the sum of a tangential and a perpendicular component $u_{\|}^{\prime }$ and $u_{⊥}^{\prime }$ in the body frame. The propulsive force is obtained by multiplying $u_{\|}^{\prime }$ and $u_{⊥}^{\prime }$, with the friction coefficients *ζ*_‖_ and *ζ*_⊥_.

To calculate $F_{x}^{\text{prop}}$, $F_{y}^{\text{prop}}$ and $\tau_{z}^{\text{prop}}$ for a given beating pattern of axoneme in the body-fixed frame, we used the classical framework of resistive-force theory (RFT), which neglects long-range hydrodynamic interactions between different parts of the flagellum as well as the inter-flagella interactions [26, 27]. In this theory, the flagellum is discretized as a set of small rod-like segments moving with velocity **u**′(*s*, *t*) in the body-frame, as illustrated in Fig 2. The propulsive force **F**^prop^ is proportional to the local center-line velocity components of each segment in parallel and perpendicular directions:

$$\begin{array}{c} \begin{array}{c} F^{\text{prop}}\left(s,t\right)=\zeta_{\|}u_{\|}^{\prime }\left(s,t\right)+\zeta_{⊥}u_{⊥}^{\prime }\left(s,t\right),\\ u_{\|}^{\prime }\left(s,t\right)=[\dot{r}\left(s,t\right).t\left(s,t\right)]t\left(s,t\right),\\ u_{⊥}^{\prime }\left(s,t\right)=\dot{r}\left(s,t\right)-u_{\|}^{\prime }\left(s,t\right), \end{array} \end{array}$$ (5)

where $u_{\|}^{\prime }$ and $u_{⊥}^{\prime }$ are the projections of the local velocity on the directions parallel and perpendicular to the axoneme. The friction coefficients in parallel and perpendicular directions are defined as *ζ*_∥_ = 4*πμ*/(ln(2*L*/*a*) + 0.5) and *ζ*_⊥_ = 2*ζ*_‖_ [26], respectively. This anisotropic hydrodynamic friction experienced by a cylindrical segment is the basis of propulsion by a flagellum. For a water-like environment with dynamic viscosity *μ* = 10^−3^ Pa s, we obtain *ζ*_‖_ ∼ 2.1 pN msec/*μ*m^2^. As *ζ*_‖_ ≠ *ζ*_⊥_, Eq (5) implies that the resulting velocity is not parallel to the propulsive force **F**^prop^. In the following, we introduce the two dimensionless quantities:

$$\begin{array}{c} \eta \equiv \frac{\zeta_{\|}}{\zeta_{⊥}}\;\;\;\text{and}\;\;\;\zeta_{⊥}^{\prime }\equiv \frac{\zeta_{⊥}}{\mu }\;. \end{array}$$ (6)

The value of *η* will be fixed to 1/2 in the rest of the text [26]. The value of $\zeta_{⊥}^{\prime }$ is determined by the geometry of the axoneme. We take for the axoneme radius a realistic value of *a* = 0.1 *μ*m and a contour length of *L* = 10 *μ*m.

Here is a brief summary of the steps in the RFT analysis: First, we translate and rotate the axoneme such that the basal end is at position (0, 0) and the local tangent vector at *s* = 0 at any time *t* is along the ***x***-axis (see Fig 1C). In this way, we lose the orientation information of the axoneme at all the time points except for the initial configuration at time *t* = 0. Second, we calculate propulsive forces and torque in the body-frame using RFT (Eq 5), and then use Eq 4 to obtain translational velocities *U*_*x*_, *U*_*y*_ as well as rotational velocity Ω_*z*_ of the axoneme. Now the infinitesimal rotational matrix can be expressed as

$$\begin{array}{c} d\Gamma \left(t\right)=(\begin{array}{ccc} \text{cos}(\Omega_{z}\left(t\right)dt) & -\text{sin}(\Omega_{z}\left(t\right)dt) & U_{x}\left(t\right)dt\\ \text{sin}(\Omega_{z}\left(t\right)dt) & \text{cos}(\Omega_{z}\left(t\right)dt) & U_{y}\left(t\right)dt\\ 0 & 0 & 1 \end{array}), \end{array}$$ (7)

which we use to update the rotation matrix as **Γ**(*t* + *dt*) = **Γ**(*t*)*d***Γ**(*t*), considering **Γ**(*t* = 0) to be the unity matrix. Having the rotation matrix at time *t*, we obtain the configuration of the axoneme at time *t* from its shape at the body-fixed frame by multiplying the rotation matrix as **r**_lab-frame_(*s*, *t*) = **Γ**(*t*)**r**_body-frame_(*s*, *t*), which can then be compared with the experimental data. Please note that **r**_lab-frame_(*s*, *t*) = (**X**_lab-frame_(*s*, *t*), **Y**_lab-frame_(*s*, *t*), 1) is an input from experimental data presenting the beating patterns in the laboratory frame.

S2 Fig in S1 File shows a comparison between the rotational and translational velocities measured directly with the experimental data presented in Fig 1 and the results obtained with RFT using the experimental beat pattern as input (as explained above). This comparison shows a semi-quantitative agreement, therefore justifying our analysis in the framework of RFT in section 3.2.

### 2.2 Drag matrix of a bead in 2D

**Fig 3:**
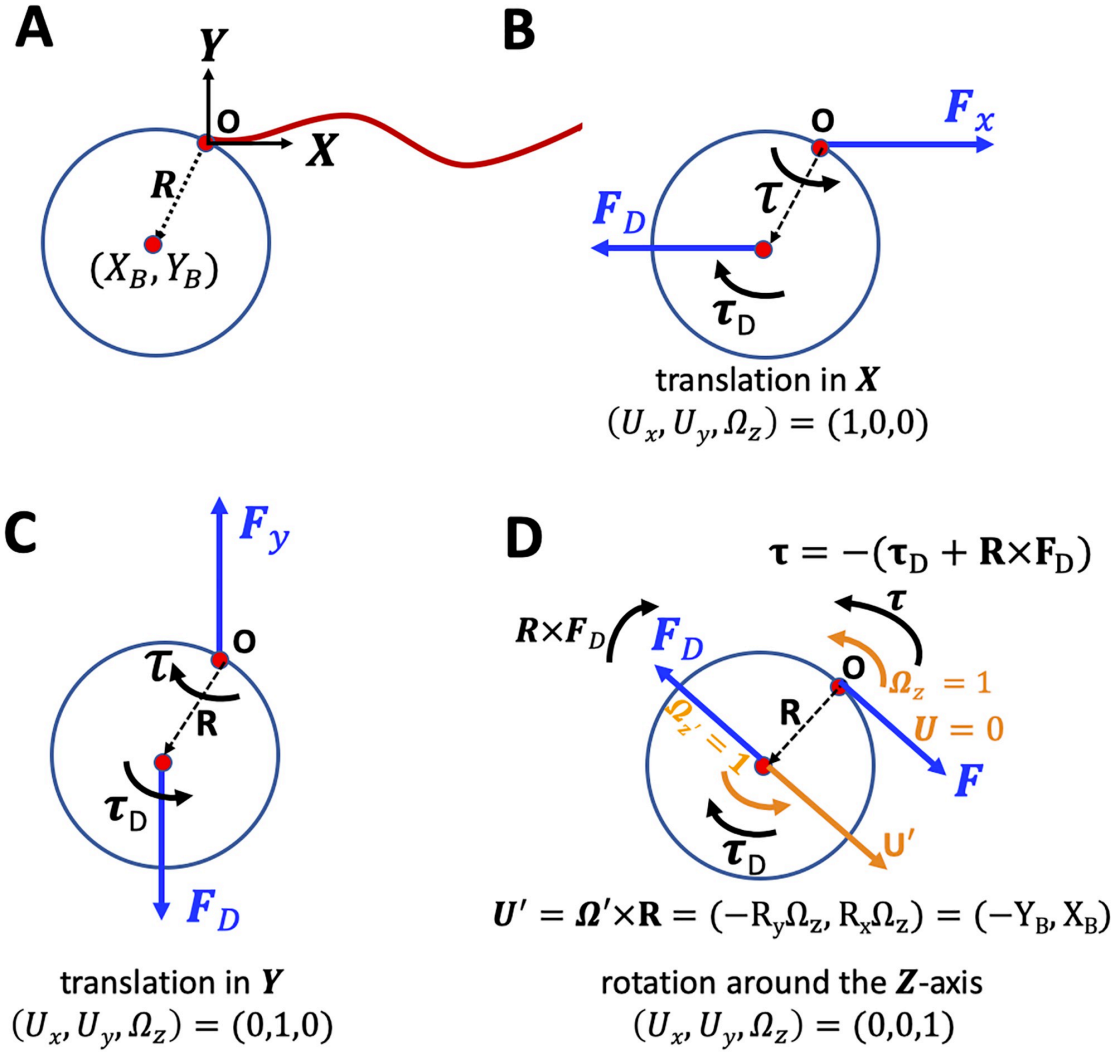
A) Definition of the swimmer-fixed frame, and illustration of the bead orientation with respect to the axoneme in 2D. The **X**-direction is given by the tangent vector at *s* = 0 (basal end). We note that *X*_*B*_ = −*R* and *Y*_*B*_ = 0 corresponds to a symmetric bead-axoneme attachment, where the tangent vector at *s* = 0 passes through the bead center. B-D) Schematic drawing of the forces and torques that counteract the hydrodynamic drag force and torque.

Let us consider the two-dimensional geometry defined in Fig 3A. Note that the origin of the swimmer-fixed frame is not at the bead center; rather it is selected to be at the bead-axoneme contact point. In general, the tangent vector at position *s* = 0 of the axoneme, which defines the **X**-axis, does not pass through the bead center located at (*X*_*B*_, *Y*_*B*_) (note that $X_{B}^{2}+Y_{B}^{2}=R^{2}$). This asymmetric bead-axoneme attachment is also observed in the experiments, as shown in Fig 1A. We emphasize that the drag force is actually a distributed force, given by *d* **f** = *σ*.*d* **A**, applied at the surface of the sphere, but symmetry implies that drag force effectively acts on the bead center. We define the translational and rotational friction coefficients of the bead as *ν*_*T*_ = 6*πα*_*t*_*μR* and *ν*_*R*_ = 8*πα*_*r*_*μR*^3^, where *μ* = 10^−3^ Pa s is the dynamic viscosity of water and factors *α*_*t*_ = 1/(1 − 9(*R*/*h*)/16 + (*R*/*h*)^3^/8) and *α*_*r*_ = 1/(1−(*R*/*h*)^3^/8) are corrections due to the fact that axonemal-based bead propulsion occurs in the vicinity of a substrate [31]. Here *R* is the bead radius (∼0.5 *μ*m), and *h* is the distance of the center of the bead to the substrate. Assuming *R*/*h* ∼ 1, we obtain *α*_*t*_ = 16/9 and *α*_*r*_ = 8/7. We now look at each component of velocity and ask what force do we need to apply to counteract the viscous force and torque?

- Translation in **X**-direction. In this case, we have (*U*_*x*_, *U*_*y*_, Ω_*z*_) = (1, 0, 0) as shown in Fig 3B. We need to apply a force in +**X**-direction to counteract the drag force as:
  $$\begin{array}{c} F_{x}=6\pi \alpha_{t}\mu R=\nu_{T}. \end{array}$$ (8)
  But we must also apply a torque in +**Z**-direction for the case illustrated in Fig 3B (where *Y*_*B*_ < 0) to prevent rotation from occurring:
  $$\begin{array}{c} \tau =-\tau_{D}=-Y_{B}F_{x}=-\nu_{T}Y_{B}, \end{array}$$ (9)
  so we get:
  $$\begin{array}{c} \left(F_{x},F_{y},\tau_{z}\right)=\left(\nu_{T},0,-\nu_{T}Y_{B}\right)U_{x}. \end{array}$$ (10)
- Translation in **Y**-direction, see Fig 3C, corresponding to (*U*_*x*_, *U*_*y*_, Ω_*z*_) = (0, 1, 0). We have *F*_*x*_ = 0 and *F*_*y*_ = + 6*πα*_*t*_*μR* = *ν*_*T*_. Note that we need to apply a negative torque, and since *X*_*B*_ < 0, we have *τ*_*z*_ = + *X*_*B*_*ν*_*T*_ which gives:
  $$\begin{array}{c} \left(F_{x},F_{y},\tau_{z}\right)=\left(0,\nu_{T},\nu_{T}X_{B}\right)U_{y}. \end{array}$$ (11)
- Rotation around **Z**-direction, see Fig 3D, corresponding to (*U*_*x*_, *U*_*y*_, Ω_*z*_) = (0, 0, 1). Before looking at the forces, let us examine the motion. The rotation $\Omega_{z}^{\prime }=1$ of the bead center around the origin *O* also generates translational velocity **U**′ = **Ω**′ × **R** = (−*R*_*y*_, *R*_*x*_)Ω_*z*_ = (−*Y*_*B*_, *X*_*B*_). Note that for *Y*_*B*_ < 0 and *X*_*B*_ < 0, we get $U_{x}^{\prime }>0$ and $U_{y}^{\prime }<0$ which is consistent. Around the center of the bead, drag exerts force and torque **F**_*D*_ and ***τ***_*D*_, as depicted in Fig 3D:
  $$\begin{array}{c} F_{D}=-v_{T}U^{\prime }=\left(\nu_{T}Y_{B},-\nu_{T}X_{B}\right),\;\;\;\;\text{and}\;\;\;\;\;\;\;{\left(\tau_{D}\right)}_{z}=-\nu_{R}. \end{array}$$ (12)
  To counteract the drag force, we must apply:
  $$\begin{array}{c} F_{D}=\left(-\nu_{T}Y_{B},\nu_{T}X_{B}\right),\;\;\;\;\text{and}\;\;\;\;\;\;\;\tau_{z}=\nu_{R}+FR=\nu_{R}+\nu_{T}R^{2}, \end{array}$$ (13)
  so we obtain (*F*_*x*_, *F*_*y*_, *τ*_*z*_) = (−*ν*_*T*_*Y*_*B*_, *ν*_*T*_*X*_*B*_, *ν*_*R*_ + *ν*_*T*_*R*^2^)Ω_*z*_, where *ν*_*T*_ = 6*πα*_*t*_*μR* and *ν*_*R*_ = 8*πα*_*r*_*μR*^3^.

Now we combine parts (i), (ii) and (iii) to obtain:

$$\begin{array}{c} (\begin{array}{c} F_{x}\\ F_{y}\\ \tau_{z} \end{array})=(\begin{array}{ccc} \nu_{T} & 0 & -\nu_{T}Y_{B}\\ 0 & \nu_{T} & \nu_{T}X_{B}\\ -\nu_{T}Y_{B} & \nu_{T}X_{B} & \nu_{R}+\nu_{T}R^{2} \end{array})(\begin{array}{c} U_{x}\\ U_{y}\\ \Omega_{z} \end{array}). \end{array}$$ (14)

For the special case of a symmetric attachment, with the center of the bead at (*X*_*B*_, *Y*_*B*_) = (−*R*, 0), Eq 14 simplifies to:

$$\begin{array}{c} (\begin{array}{c} F_{x}\\ F_{y}\\ \tau_{z} \end{array})=(\begin{array}{ccc} \nu_{T} & 0 & 0\\ 0 & \nu_{T} & -\nu_{T}R\\ 0 & -\nu_{T}R & \nu_{R}+\nu_{T}R^{2} \end{array})(\begin{array}{c} U_{x}\\ U_{y}\\ \Omega_{z} \end{array}). \end{array}$$ (15)

Note that Eqs 14 and 15 present the forces and torque exerted by the bead on the fluid which has opposite sign of the forces generated by the fluid on the bead, so the drag matrix of the bead **D**_*B*_ is given by:

$$\begin{array}{c} D_{B}=(\begin{array}{ccc} -\nu_{T} & 0 & \nu_{T}Y_{B}\\ 0 & -\nu_{T} & -\nu_{T}X_{B}\\ \nu_{T}Y_{B} & -\nu_{T}X_{B} & -\nu_{R}-\nu_{T}R^{2} \end{array}). \end{array}$$ (16)

The general form of the drag matrix in 3D is derived in the supplemental information.

### 2.3 Drag matrix of an axoneme in 2D

Since the axoneme beats over time, its drag matrix, which relates force and velocity, is time-dependent:

$$\begin{array}{c} D_{A}=(\begin{array}{ccc} a_{11}\left(t\right) & a_{12}\left(t\right) & a_{13}\left(t\right)\\ a_{21}\left(t\right) & a_{22}\left(t\right) & a_{23}\left(t\right)\\ a_{31}\left(t\right) & a_{32}\left(t\right) & a_{33}\left(t\right) \end{array}). \end{array}$$ (17)

Assume the motion is 2D, and consider the axonemal shapes at successive time points are given in the body frame as **r**_body-frame_(*s*, *t*) = (**X**_body-frame_(*s*, *t*), **Y**_body-frame_(*s*, *t*), 1). This can be either an input from the experimental data (see Fig 1F) or a predefined waveform of a model axoneme, as introduced in Section 3.1. The experimental shapes shown in Fig 1 were recorded with a high time resolution of 1000 Hz [14], and translated and rotated such that the tangent vector at *s* = 0 is along the *X*-axis. Factoring out an overall rotation and translation in the laboratory frame allows us to focus on the shape deformation of the axoneme.

To obtain the elements of the drag matrix for a given axonemal shape at time *t*, we work in the framework of RFT and follow the same procedure as in the case of a bead, as described in Sec. 2.2:

- Global translation of the axoneme in **X**-direction. In this case, we have (*U*_*x*_, *U*_*y*_, Ω_*z*_) = (1, 0, 0), which using Eq 5 and the given axonemal shape at time *t*, we first obtain the tangential and perpendicular velocity components for each cylindrical segment of the axoneme. Second, in the framework of RFT, we calculate the corresponding elemental force *dF* = (*dF*_*X*_(*s*, *t*), *dF*_*Y*_(*s*, *t*)) and torque *dτ*_*Z*_(*s*, *t*) = **r**_body-frame_(*s*, *t*) × *dF* exerted by each cylindrical segment of the axoneme on the fluid to counteract the drag force. Third, to obtain the drag elements *a*_11_, *a*_21_ and *a*_31_, we integrate the elemental force and torque over the whole contour length of axoneme to calculate the total force and torque exerted by the axoneme on the fluid to counteract the drag. The fluid drag force exerted on the axoneme has an opposite sign, thus:
  $$\begin{array}{c} a_{11}=-\int_{0}^{L}dF_{X}\left(s,t\right)\;\;\;,\;\;\;\;a_{21}=-\int_{0}^{L}dF_{Y}\left(s,t\right),\;\;\;\text{and}\;\;\;\;a_{31}=-\int_{0}^{L}d\tau_{Z}\left(s,t\right). \end{array}$$ (18)
- Global translation of the axoneme in **Y**-direction. In this case, we have (*U*_*x*_, *U*_*y*_, Ω_*z*_) = (0, 1, 0). The matrix elements *a*_12_, *a*_22_ and *a*_32_ are obtained as in (i).
- Global rotation of the axoneme around **Z**-direction, corresponding to (*U*_*x*_, *U*_*y*_, Ω_*z*_) = (0, 0, 1). We note that the rotation of the axoneme around the origin *O* (see Fig 3A) with **Ω** = (0, 0, Ω_*z*_), also generates translational velocity components as **Ω** × **r**_body-frame_(*s*, *t*) = (−**Y**_body-frame_(*s*, *t*), **X**_body-frame_(*s*, *t*)), that should be taken into account while using Eq 5 to obtain the force and the torque that the axoneme exerts on the fluid to counteract the drag. Similar to Eq 18, we can now calculate the drag elements *a*_13_, *a*_23_ and *a*_33_.

## 3 Results

### 3.1 Analytical approximations of rotational and translational velocities of an axonemally-propelled bead

The waveform of the axoneme is complex, and involves a combination of several components [18, 20, 22, 32]. In this first subsection, we begin by discussing a simplified waveform, in order to understand the elementary aspects of the propulsion of the bead. In practice, we approximate the waveform of the axoneme as a superposition of traveling wave component, with amplitude *C*_1_, and a circular arc with mean curvature *C*_0_ [18, 22]:

$$\begin{array}{c} C\left(s,t\right)\approx C_{0}+C_{1}\text{cos}\left(\omega_{0}t-ks\right), \end{array}$$ (19)

where *ω*_0_ = 2*πf*_0_, *k* = 2*π*/λ is the wave number. For our exemplary axoneme in Fig 1A, following the method described in Ref. [16], we calculate the wavelength to be λ ∼ 11.34 *μ*m, which is ∼34% larger than the axonemal contour length *L* ∼ 8 *μ*m. The approximate waveform given by Eq 19 allows us to obtain explicit expressions for the propulsion velocity of the cargo. This expression, however, neglects a small backwards wave component, propagating from tip-to-base of the form $C_{1}^{\prime }\text{cos}\left(\omega_{0}t+ks\right)$ [22], along with components with wave numbers equal to *n* × *k*, where *n* is an integer > 1. The results of our analysis of beating axonemes [22] show that back-propagating wave component is about 5–10 times smaller than the main base-to-tip wave. For simplicity, this small component is neglected in this subsection. The analysis presented in Subsection 3.2, based on numerical simulations with the precise waveform of the axonemes determined experimentally [14], qualitatively validates the approach presented here.

To obtain analytical expressions for the mean translational and rotational velocities of the swimmer, we used RFT, as presented in the Materials and Methods section. As a further simplification, we neglect in this section the difference between the wavelength of the beat pattern, λ, and the size of the axoneme, *L*, and assume in the following *L* = λ. We determine the propulsion velocity up to the first order in *C*_0_, and the second order in *C*_1_.

#### 3.1.1 Symmetric bead-axoneme attachment

Let us first consider the example of an axoneme attached symmetrically from the basal side to a bead, so that the tangent vector at *s* = 0 passes through the bead center (*X*_*B*_ = −*R*, *Y*_*B*_ = 0, see Fig 3B). We determine the dependence of the translational and rotational velocities of the swimmer on the dimensionless bead radius *r* = *R*/*L*, with the simplified waveform of the axoneme given by Eq 19 and impose the force-free and torque-free conditions in 2D. The drag matrix of the bead is given by Eq 16, with *Y*_*B*_ = 0 and *X*_*B*_ = −*R*. The drag matrix of the axoneme is calculated as described in Sec. 2.2.

We approximate analytically the averaged angular and linear velocities in the swimmer-fixed frame, as defined in Fig 1C, and we determine the propulsion velocities up to first order in *C*_0_, and to second order in *C*_1_:

$$\begin{array}{cc} & \frac{\left\langle \Omega_{z}\right\rangle }{\omega_{0}}\approx \beta_{1}\left(r,\zeta_{⊥}^{\prime },\eta \right)C_{0}C_{1}^{2}, \end{array}$$ (20)

$$\begin{array}{cc} & \frac{\left\langle U_{x}\right\rangle }{L\omega_{0}}\approx \beta_{2}\left(r,\zeta_{⊥}^{\prime },\eta \right)C_{1}^{2}, \end{array}$$ (21)

$$\begin{array}{cc} & \frac{\left\langle U_{y}\right\rangle }{L\omega_{0}}\approx \beta_{3}\left(r,\zeta_{⊥}^{\prime },\eta \right)C_{0}C_{1}^{2}. \end{array}$$ (22)

**Fig 4:**
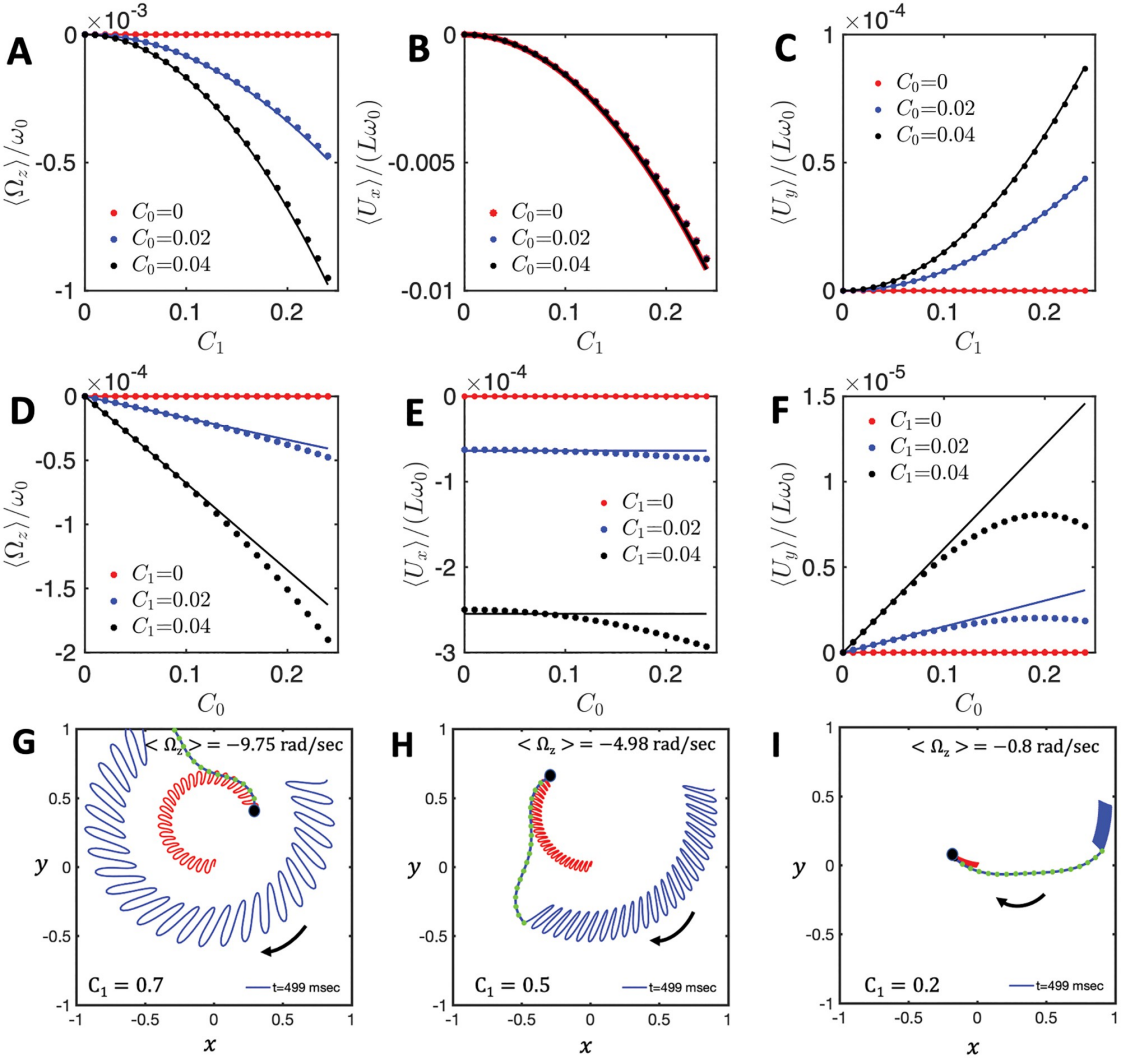
A-F) Comparison between the analytical approximations for the rotational and translational velocities, Eqs 20–22 (solid lines), and the results of numerical simulations (dots) for bead radius of *R* = 0. G-I) Numerical simulations performed with the simplified waveform to show the effect of *C*_1_. A bead of radius *R*, with *r* = *R*/*L* = 0.1, is attached symmetrically to a model flagellum. At a fixed value of the static curvature, *C*_0_ = 0.2, the mean rotational velocity decreases as the amplitude of dynamic mode *C*_1_ decreases from G) *C*_1_ = 0.7, to H) *C*_1_ = 0.5 and further to I) *C*_1_ = 0.2.

The functions *β*_1_, *β*_2_ and *β*_3_ depend on the dimensionless quantities $\zeta_{⊥}^{\prime }$ and *η*, defined by Eq 6, and on *r* = *R*/*L*. The explicit expressions are presented in the supplemental information, Eqs. S16-S18. The results in the absence of the bead simplifies to:

$$\begin{array}{cc} & \frac{\left\langle \Omega_{z}\right\rangle }{\omega_{0}}\approx -0.42C_{0}C_{1}^{2}, \end{array}$$ (23)

$$\begin{array}{cc} & \frac{\left\langle U_{x}\right\rangle }{L\omega_{0}}\approx -0.16C_{1}^{2}, \end{array}$$ (24)

$$\begin{array}{cc} & \frac{\left\langle U_{y}\right\rangle }{L\omega_{0}}\approx +0.038C_{0}C_{1}^{2}\;. \end{array}$$ (25)

which are previously discussed in Refs. [19, 20]. The corresponding dependence on *C*_0_ and *C*_1_ is shown in Fig 4A–4F as full lines. We note that Eqs 20–22, in the absence of intrinsic curvature *C*_0_ = 0, predict that the axoneme swims in a straight path with 〈*U*_*y*_〉 = 0, 〈*U*_*x*_〉 proportional to the square of traveling wave component *C*_1_ [33–35] and the mean rotational velocity 〈Ω_*z*_〉 vanishes (see the solid red line in Fig 4A and S3A Fig in S1 File).

In order to verify the quality of our analytical approximations, we also determined the motion of the swimmer numerically using RFT, starting from the simplified waveform given in Eq 19 and *r* = 0. The corresponding results are shown by the circular symbols in Fig 4A–4F. The comparison between numerical simulations and the full analytical approximations presented in Eqs. S16-S18, shows a very good agreement at small values of *C*_0_ and *C*_1_, with deviations at larger values. In addition, three exemplary trajectories (*r* = 0.1), determined from RFT, are shown in Fig 4G–4I. The corresponding averaged rotational velocity 〈Ω_*z*_〉 of the model swimmer is proportional to the square of the traveling wave component *C*_1_.

**Fig 5:**
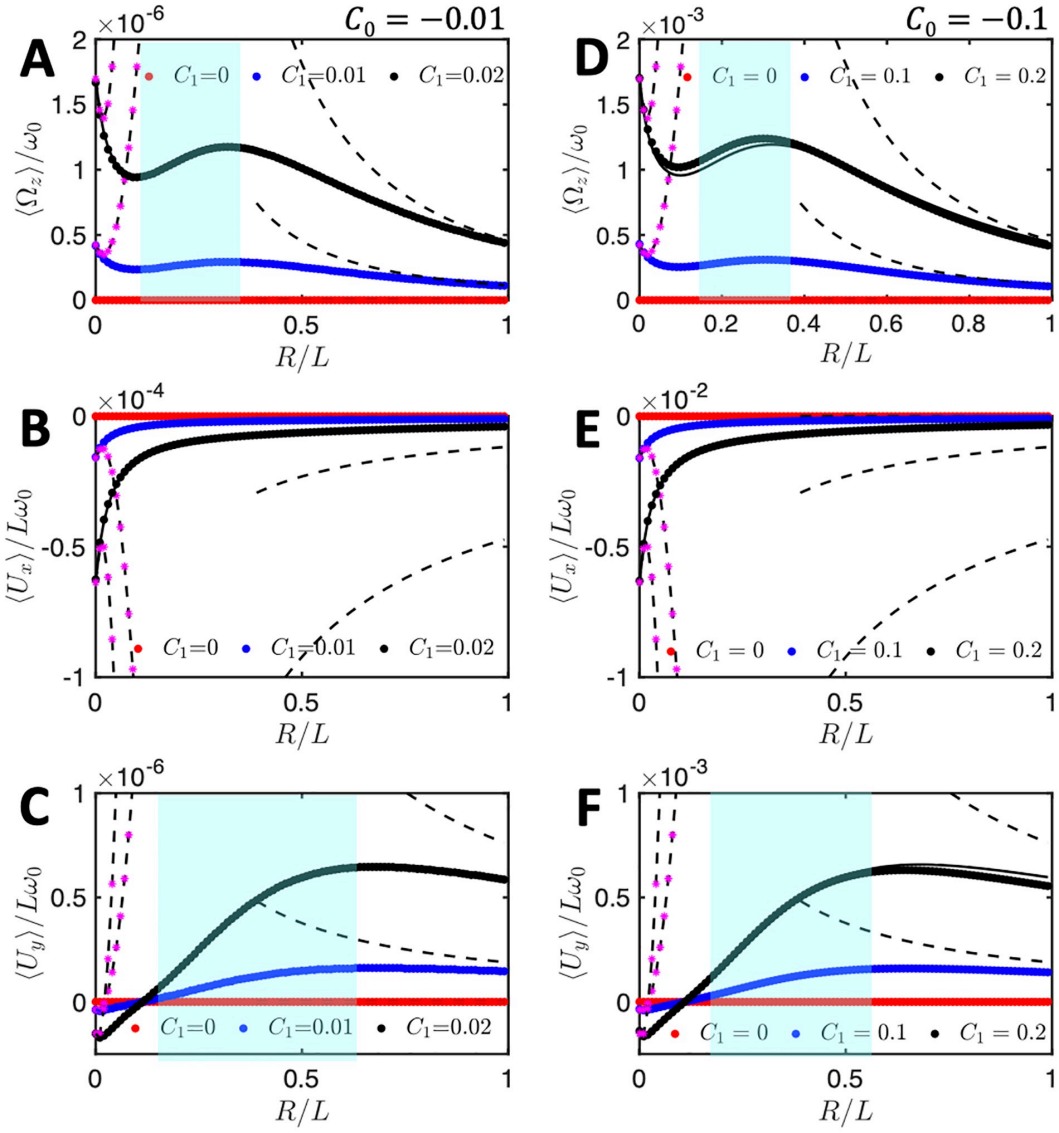
Anomalous flagella-based propulsion speed of a symmetrically attached bead as a function of its dimensionless radius *r* = *R*/*L*. Contrary to expectations, in the region highlighted in cyan, the mean translational and rotational velocities increase with increasing the bead radius. Analytical approximations (continuous lines calculated from Eqs 20–22) and simulations (dotted points) are performed at different values of *C*_1_, while the intrinsic curvature of the axoneme is fixed at *C*_0_ = −0.01 in (A-C), and *C*_0_ = −0.1 in (D-F). The black dashed curves show the trend expected in the limit of large bead radius (*r* = *R*/*L* > 1), as presented in Eqs 26–28. The black dashed lines with stars in magenta illustrate the opposite limit of the small *r*, as given in Eqs 29–31.

To investigate the dependency of the mean translational and rotational velocities of our model swimmer on the bead size, *r*, Fig 5 shows the dependence of 〈Ω_*z*_〉/*ω*_0_ (A and D), 〈*U*_*x*_〉/(*Lω*_0_) (B and E) and 〈*U*_*y*_〉/(*Lω*_0_) (C and F), predicted by Eqs 20–22; see the full lines. We also performed numerical simulations at different values of the bead radii, see the circular symbols. As shown in Fig 5, there is a very good agreement between our numerical simulations and analytical approximations at small values of *C*_1_ (panels A-C) but deviations appear at larger values (panels D-F). Remarkably, while 〈*U*_*x*_〉 decreases monotonically with the bead radius *r* = *R*/*L*, both 〈Ω_*z*_〉 and 〈*U*_*y*_〉 exhibit a non-monotonic dependence. This behavior is counter-intuitive: the drag exerted by the fluid on the sphere increases with the size of the bead, which in turn increases dissipation. Based on this general remark, one expects the velocity of the swimmer to go down when *r* increases. We nevertheless notice that not all three components of velocity may increase when *r* increases.

To gain more insight on this anomalous behavior, we determined the asymptotic expressions of Eqs 20–22 in two opposite limits of small and large bead radii. The corresponding dependence is shown by the dashed lines in Fig 5. In the limit of very large bead radius, *R* ≫ *L* (small 1/*r*), we obtain a dependence of 〈*U*_*x*_〉 and 〈*U*_*y*_〉 as *r*^−1^, and of 〈Ω_*z*_〉 as *r*^−2^ (up to the higher order corrections):

$$\begin{array}{c} \frac{\left\langle \Omega_{z}\right\rangle }{\omega_{0}}\approx \frac{21C_{0}C_{1}^{2}\left(\eta -1\right)\zeta_{⊥}^{\prime }r^{-2}}{64}, \end{array}$$ (26)

$$\begin{array}{c} \frac{\left\langle U_{x}\right\rangle }{L\omega_{0}}\approx \frac{7C_{1}^{2}\left(\eta -1\right)\zeta_{⊥}^{\prime }r^{-1}}{36864\pi^{2}}\left(576\pi -7\left(6\eta +5\right)\zeta_{⊥}^{\prime }r^{-1}\right), \end{array}$$ (27)

$$\begin{array}{c} \frac{\left\langle U_{y}\right\rangle }{L\omega_{0}}\approx -\frac{7C_{0}C_{1}^{2}\zeta_{⊥}^{\prime }r^{-1}}{36864\pi^{2}}(35\pi \left(\eta -1\right)\left(4\eta +21\right)\zeta_{⊥}^{\prime }r^{-1}\\ +720\left(3\eta -1\right)r^{-1}-96\pi^{2}\left(\eta \left(9r^{-1}+30\right)-11r^{-1}-30\right)). \end{array}$$ (28)

In the opposite limit of small *r* (i.e. *R* ≪ *L*), up to the second order in *r*, we obtain with the realistic value of $\zeta_{⊥}^{\prime }\approx 4.33$:

$$\begin{array}{c} \frac{\left\langle \Omega_{z}\right\rangle }{\omega_{0}}\approx -0.42C_{0}C_{1}^{2}\left(1-19.15r+513.77r^{2}\right), \end{array}$$ (29)

$$\begin{array}{c} \frac{\left\langle U_{x}\right\rangle }{L\omega_{0}}\approx 0.16C_{1}^{2}\left(1-29.85r+960.23r^{2}\right), \end{array}$$ (30)

$$\begin{array}{c} \frac{\left\langle U_{y}\right\rangle }{L\omega_{0}}\approx 0.038C_{0}C_{1}^{2}\left(1+39.8r-3941.65r^{2}\right). \end{array}$$ (31)

The black dashed lines (with and without stars in pink color) in Fig 5 show the corresponding asymptotic behavior. The limiting behavior is valid only for very small values of *r*. It nevertheless qualitatively captures the non-monotonous trend, observed at intermediate values of *r*. The transition from the *r*^2^ dependence at small values of bead radius to the *r*^−2^-trend at large values of *r* provides a qualitative explanation for the non-monotonous behavior, observed for 〈Ω_*z*_〉 and 〈*U*_*y*_〉.

#### 3.1.2 The sideways bead-axoneme attachment contributes to the rotational velocity of the swimmer

In the experiments in Ref. [14], it was frequently observed that the bead-axoneme attachment was asymmetric, i.e. the tangent vector of the axoneme at *s* = 0 does not pass through the bead center. This case is schematically illustrated in Fig 3A, where it results in a value of *Y*_*B*_ ≠ 0. Interestingly, our analytical approximations and simulations show that this asymmetric bead-axoneme attachment is enough to rotate the axoneme, so the presence of the static curvature or the second harmonic is not necessary for rotation to occur.

For this analysis, we consider the 2D geometry where the center of the bead is at position *X*_*B*_ and *Y*_*B*_, measured with respect to the coordinate system defined at the bead-axoneme contact point (Figs 1C and 3A). We will use here the dimensionless coordinates *x*_*B*_ = *X*_*B*_/*L* and *y*_*B*_ = *Y*_*B*_/*L* (note that $x_{B}^{2}+y_{B}^{2}={\left(R/L\right)}^{2}=r^{2}$). The drag matrix of the bead is given by Eq 16, where we specify the value of *Y*_*B*_ ≠ 0 corresponding to the asymmetric attachment. The beating of the axoneme is described by Eq 19 in terms of a traveling wave component *C*_1_ and intrinsic curvature *C*_0_. Similar to the case of a symmetric bead-axoneme attachment in Section 3.1.1, we calculate the mean rotational and translational velocities of an axonemally-driven bead by combining the drag matrix of the bead and the axoneme (see Materials and methods). The results up to the leading order in *C*_0_ and *C*_1_ can be expressed as:

$$\left\langle \Omega_{z}\right\rangle /\omega_{0}\approx (\alpha_{1}\left(\eta ,\zeta_{⊥}^{\prime },y_{b},r\right)+\alpha_{1}^{\prime }\left(\eta ,\zeta_{⊥}^{\prime },y_{b},r\right)C_{0})C_{1}^{2},$$ (32)

$$\left\langle U_{x}\right\rangle /L\omega_{0}\approx (\alpha_{2}\left(\eta ,\zeta_{⊥}^{\prime },y_{b},r\right)+\alpha_{2}^{\prime }\left(\eta ,\zeta_{⊥}^{\prime },y_{b},r\right)C_{0})C_{1}^{2},$$ (33)

$$\left\langle U_{y}\right\rangle /L\omega_{0}\approx (\alpha_{3}\left(\eta ,\zeta_{⊥}^{\prime },y_{b},r\right)+\alpha_{3}^{\prime }\left(\eta ,\zeta_{⊥}^{\prime },y_{b},r\right)C_{0})C_{1}^{2}$$ (34)

where *η* and $\zeta_{⊥}^{\prime }$ are defined by Eq 6, *r* = *R*/*L*, and *y*_*b*_ = *Y*_*b*_/*L*. When the attachment is symmetric (*y*_*B*_ = 0), symmetry considerations impose that:

$$\begin{array}{c} \alpha_{1}\left(\eta ,\zeta_{⊥}^{\prime },y_{b}=0,r\right)=0\;\;\;\text{and}\;\;\;\alpha_{3}\left(\eta ,\zeta_{⊥}^{\prime },y_{b}=0,r\right)=0\;, \end{array}$$ (35)

which expresses the fact that in the absence of *C*_0_, both 〈Ω_*z*_〉 and 〈*U*_*y*_〉 are zero. This is also consistent with previous expressions of 〈Ω_*z*_〉 and 〈*U*_*y*_〉 when the attachment is symmetric, see Eqs 26,28,29 and 31. In contrast, when the attachment is not symmetric (*y*_*B*_ ≠ 0), the coefficients $\alpha_{1}\left(\eta ,\zeta_{⊥}^{\prime },y_{B},r\right)$ and $\alpha_{3}\left(\eta ,\zeta_{⊥}^{\prime },y_{B},r\right)$ become non zero, which implies that the system rotates (〈Ω_*z*_〉≠0) and has a nonzero velocity component 〈*U*_*y*_〉≠0 even when *C*_0_ = 0. This clearly shows the importance of the asymmetric bead-axoneme attachment.

**Fig 6:**
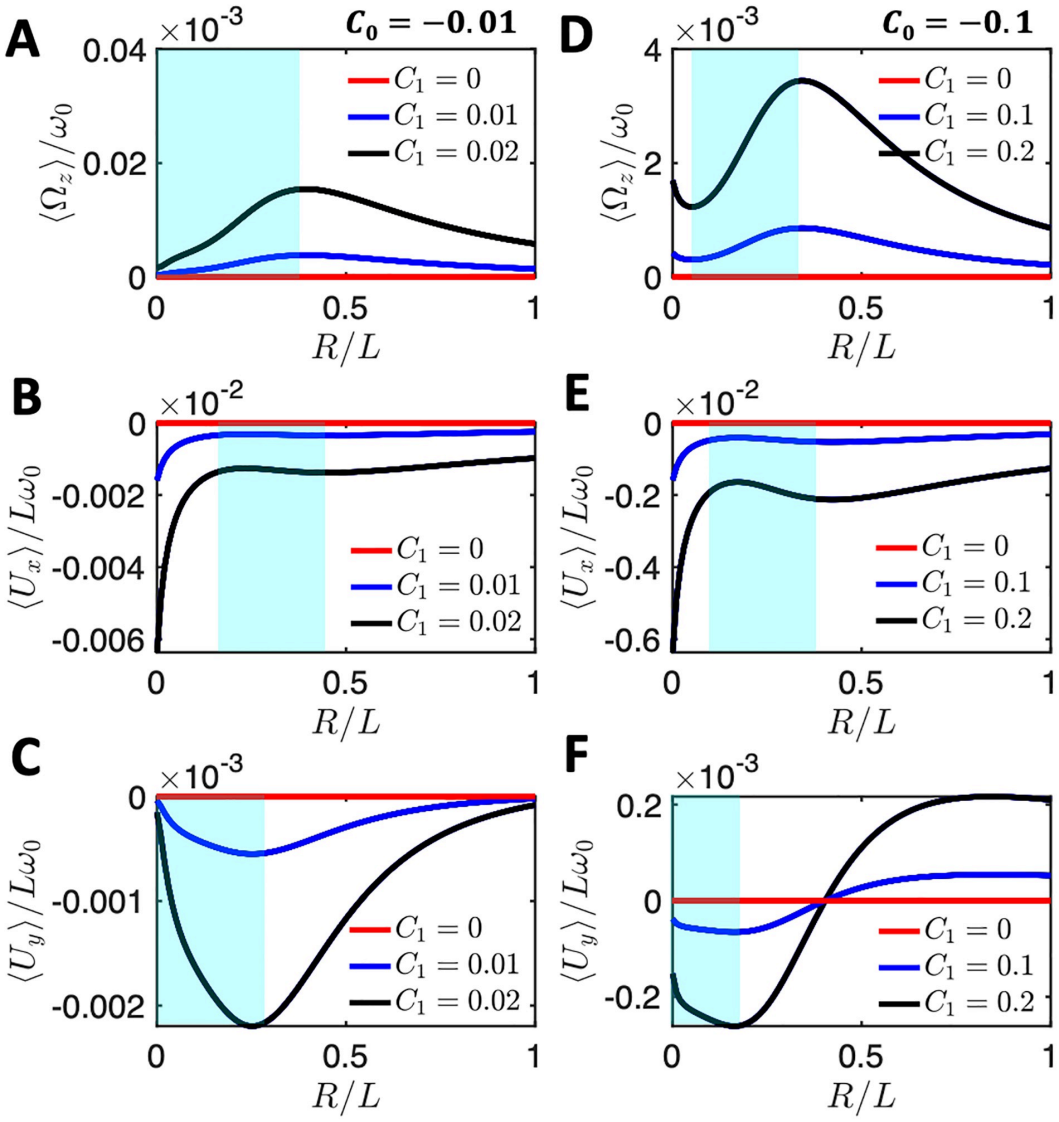
The mean rotational and translational velocities of a bead which is asymmetrically attached to an axoneme with *y*_*b*_ = −*r* and *x*_*b*_ = 0 for different values of *C*_1_, as a function of the dimensionless ratio *r* = *R*/*L*. The mean curvature *C*_0_ is −0.01 in panels A-C and −0.1 for panels D-F. For a sketch of the bead-axoneme attachment geometry see Fig 7B.

**Fig 7:**
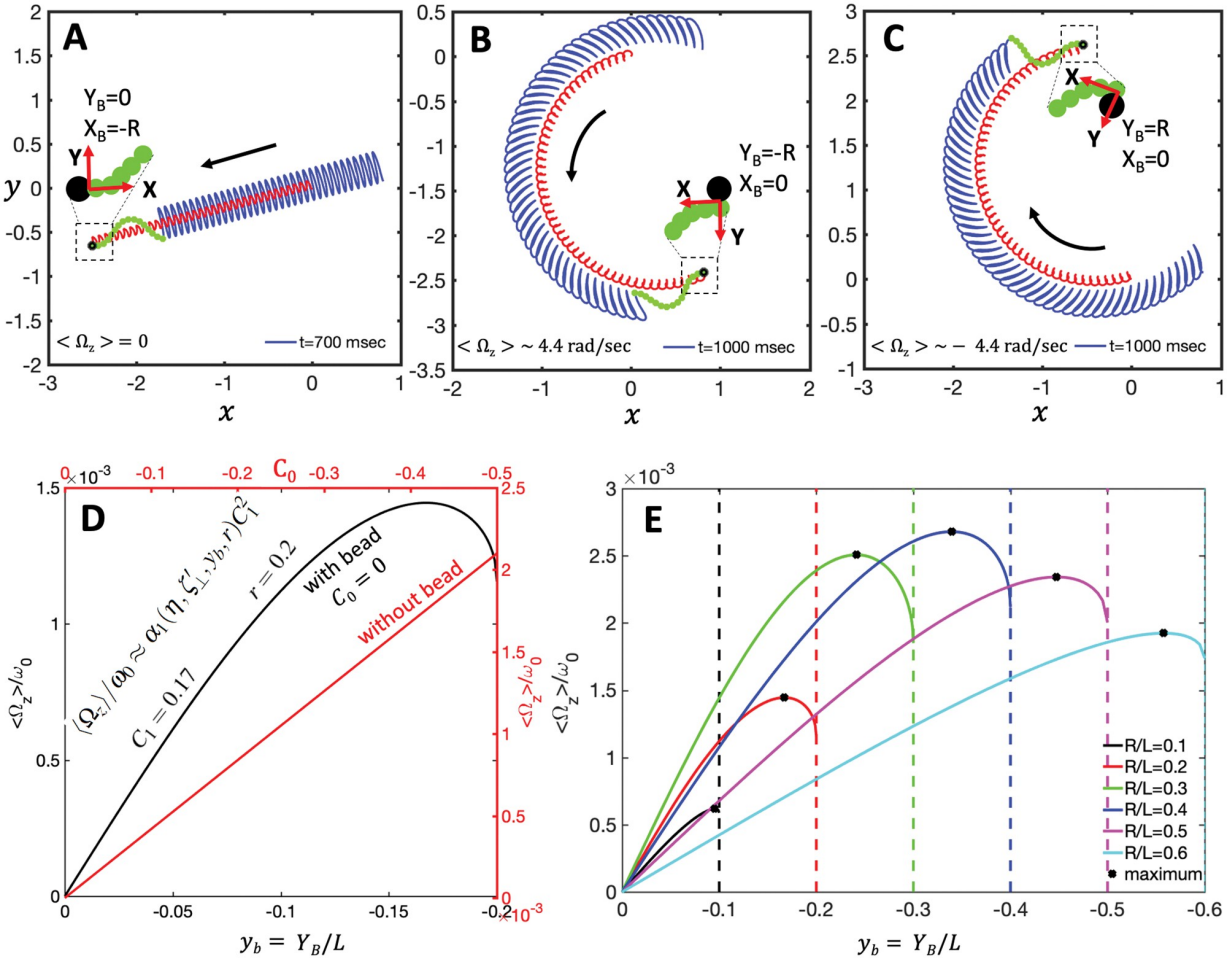
A-C) Asymmetric versus symmetric bead attachment to an axoneme with a beat pattern consisting only of the traveling wave component *C*_1_ (*C*_0_ = 0 in Eq 19). While the axoneme in panel A, to which the bead is symmetrically attached, swims on a straight path (S3 Video), the axonemes in panel B (S4 Video) and C (S5 Video), with an asymmetric bead attachment, swim on curved paths. D) Comparison of the effect of the asymmetric bead attachment (in black) as a function of *y*_*b*_ = *Y*_*B*_/*L* versus the effect of the intrinsic curvature *C*_0_ (in red) on 〈Ω_*z*_〉. E) The averaged angular velocity 〈Ω_*z*_〉 changes non-monotonically with *y*_*b*_ for different bead radii. *X*_*B*_ and *Y*_*B*_ are the coordinates of the bead center in the swimmer-fixed reference frame. Parameters are *η* = 0.5, $\zeta_{⊥}^{\prime }=4.33$ and *C*_1_ = 0.1.

This is illustrated by Fig 6, which shows the translational and rotational velocities of the swimmer for different values of *C*_0_ and *C*_1_ and a sideways bead attachment of *x*_*b*_ = 0 and *y*_*b*_ = −*r*, as shown schematically in Fig 7B. The full analytic forms of *α*_*i*_ and $\alpha_{i}^{\prime }$ (*i* = 1, 2, 3) are very long and not particularly informative, so we only present closed form expressions for the coefficients *α*_*i*_, defined by Eqs 32–34, when *C*_0_ = 0 in the supporting information, see Section 3. In particular, Eq 22 shows the closed form of *α*_1_. For *α*_2_ and *α*_3_, Eqs. S23- S24) show the expressions for the even more restricted case where *C*_0_ = 0 and *y*_*b*_ = −*r*.

We also performed numerical simulations to study the effect of an asymmetric bead-axoneme attachment on the swimming motion (see Fig 7). In these simulations, the model axoneme has only the traveling wave component *C*_1_ and it swims in a straight path if the bead is attached symmetrically i.e. when *y*_*B*_ = 0; see Fig 7A and S3 Video. An asymmetric bead-axoneme attachment causes the axoneme to rotate, as illustrated by Fig 7B and 7C; see also the S4 and S5 Videos. Thus, consistent with Eqs 32–34, our numerical simulations show that an asymmetric attachment of the axoneme to the bead causes rotation of the swimmer, even in the absence of static curvature (*C*_0_ = 0).

It is interesting to compare the effect of the intrinsic curvature (Eq. S16 with *r* = 0) versus asymmetric bead attachment (Eq 32 with *C*_0_ = 0) on the rotational velocity of the swimmer. To this end, Fig 7D presents a comparison between the influence on the rotational velocity, 〈Ω_*z*_〉, of an asymmetric attachment, as a function of *y*_*B*_ (lower horizontal and left vertical axes; black line), and of intrinsic curvature, *C*_0_ (upper horizontal and right vertical axes; red line). The results presented in Fig 7D show that the contribution of the asymmetry in the attachment to 〈Ω_*z*_〉 is comparable to that of the intrinsic curvature, *C*_0_. We also observe that, as shown in Fig 7E, the maximum rotational velocity is found at values of *y*_*b*_ close to −*r*.

### 3.2 Analysis with the experimental waveform

To confirm that the predictions of the previous subsection of the existence of an anomalous propulsion regime and of a rotation induced by asymmetric cargo attachment is general and not limited to the very simplified waveform given by Eq 19, we also used the experimental beat patterns and performed RFT simulations to compute mean translational and rotational velocities of an axonemally-propelled bead for both asymmetric and symmetric bead-axoneme attachment and various bead radii.

For this purpose, we used the experimental beat pattern shown in Fig 3A of Ref. [14] (see S6 Video). As explained in our recent studies [14, 22], we performed principal component analysis (PCA) of the experimental beat patterns, and then, decomposed the eigenmodes as Fourier series. Our analysis reveals that the traveling curvature waves can be decomposed into a static component *C*_0_ and a leading traveling wave component of amplitude *C*_1_ that coexist with standing waves at the traveling wave number and at multiples of this wave number (higher harmonics). This Fourier analysis of the experimental data indeed justifies the decomposition of the waveform as defined in Eq 19 for our analytical study.

**Fig 8:**
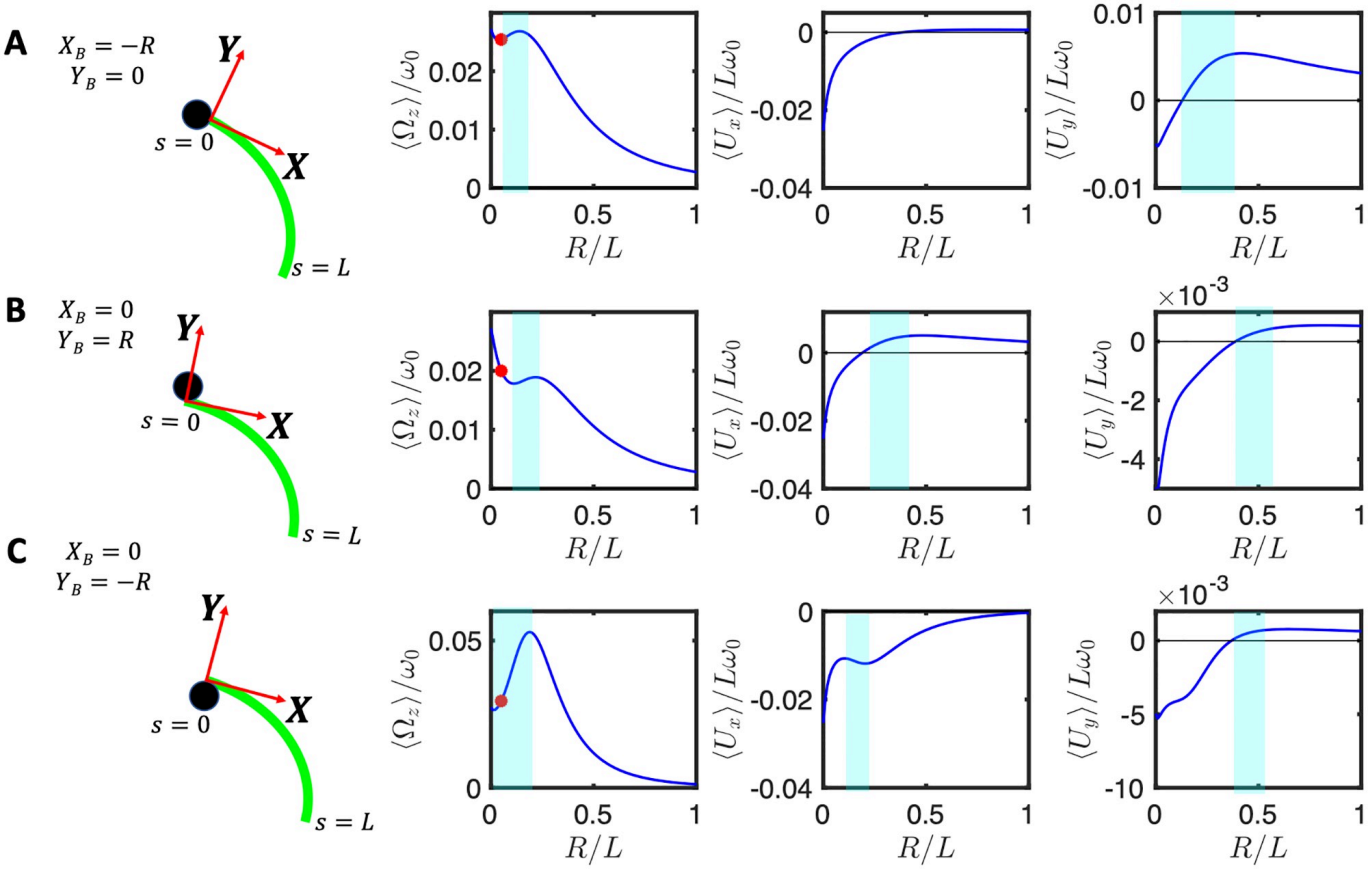
Experimental beat pattern presented in Ref. [14] (see S6 Video) are used to calculate rotational and translational velocities of an axonemally-propelled bead attached (A) symmetrically at *Y*_*B*_ = 0, *X*_*B*_ = −*R*, (B) asymmetrically at *Y*_*B*_ = *R*, *X*_*B*_ = 0 (C) asymmetrically at *Y*_*B*_ = −*R*, *X*_*B*_ = 0. Anomalous propulsion regimes are highlighted in cyan color. Red circles mark the experimental bead size of *R*/*L*∼0.05 and the corresponding rotational velocities. Note that the trend observed in panel A is consistent with the trend predicted by our analytical calculations with a simplified waveform for a symmetric bead-axoneme attachment as shown in Fig 5.

The results of our determination of the mean translational and rotational velocities as a function of the bead radius is shown in Fig 8. Although the results are quantitatively different from those in Fig 5 obtained with the simplified waveform given by Eq 19, the variations of 〈Ω_*z*_〉 and one of the translational velocity show a non-monotonic dependence on *r* = *R*/*L* for the case when the attachment is symmetric, as shown in Fig 8A. The results obtained with the experimentally realistic waveform are therefore qualitatively consistent with those obtained with the simplified waveform, Eq 19.

In the case of an asymmetric attachment, depending on the sign of the static curvature of the axoneme *C*_0_ and the position at which the bead is attached, one may observe an increase or decrease in the overall mean rotational velocities. For the axoneme in Fig 8, *C*_0_ is negative and the sideways bead attachment at *Y*_*B*_ = *R* (Fig 8B) acts against the rotation induced by the intrinsic curvature *C*_0_. The opposite happens in Fig 8C where the bead attachment at *Y*_*B*_ = −*R* amplifies the rotational velocity of the axoneme. We also note that the anomalous propulsion regime is more pronounced in panel C where the bead is attached sideways at *Y*_*B*_ = −*R*.

Furthermore, in the right panels of Fig 8A–8C, the measured values of 〈Ω_*z*_〉/*ω*_0_ using RFT at the experimental bead size of *R* = 0.5 *μ*m (so the ratio *R*/*L* = 0.05), are indicated by red circles. We note that in this experiment (see S6 Video) the axoneme globally rotates around 2*π* in the time interval of ∼650 msec, and with *f*_0_ = 38.21 Hz, results to 〈Ω_*z*_〉/*ω*_0_∼0.04, which is larger than the measured RFT values of 0.025, 0.020 and 0.029, corresponding to different bead-axoneme attachment geometries in panels A-C, respectively. Overall, we observe a semi-quantitative agreement between the RFT predicted and the experimentally measured values of 〈Ω_*z*_〉/*ω*_0_.

**Fig 9:**
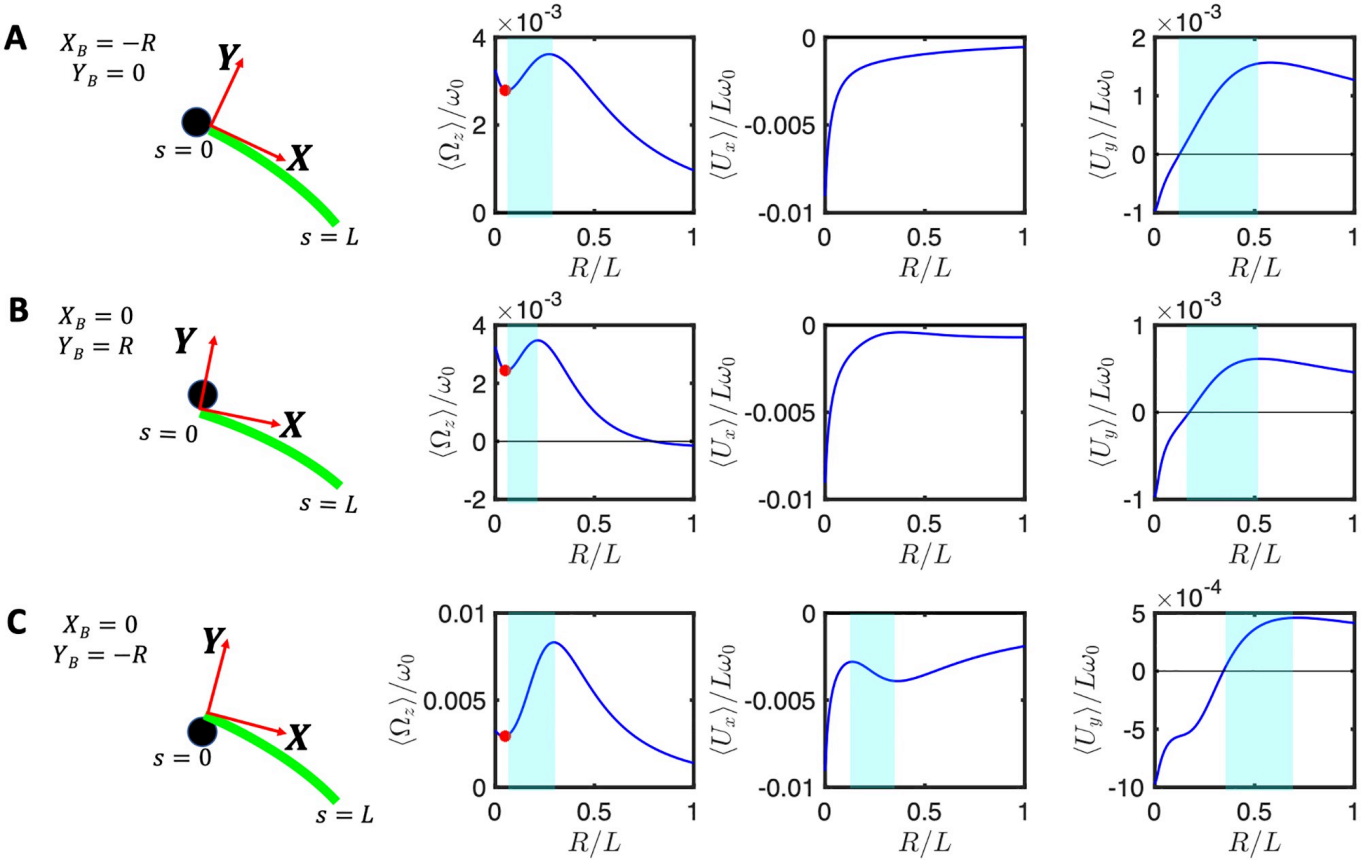
Experimental beat pattern reported in Ref. [14] with 0.1 mM [Ca^2+^] and [ATP] = 80 *μ*M (see S7 Video) are used to calculate the mean rotational and translational velocities of an axonemally-driven bead attached (A) symmetrically at *Y*_*B*_ = 0, *X*_*B*_ = −*R* (B) asymmetrically at *Y*_*B*_ = *R*, *X*_*B*_ = 0, and (C) asymmetrically at *Y*_*B*_ = −*R*, *X*_*B*_ = 0. Anomalous propulsion regimes are highlighted in cyan color. Red circles mark the experimental bead radius of *R*/*L* = 0.05 and the corresponding values of 〈Ω_*z*_〉/*ω*_0_ (see S7 Video). Note that the general trend in panel A is consistent with the analytical analysis presented in Fig 5 for a symmetric bead attachment.

An important conclusion in Ref. [14] is that at higher calcium concentration, the mean curvature *C*_0_ is strongly reduced, leading to a strong reduction of 〈Ω_*z*_〉. For this reason, we also considered the beat patterns from the experiment in Fig 3D of Ref. [14] at higher calcium concentration (S7 Video), to study the influence of the flagellar waveform on the propulsion of the swimmer, both with symmetric and asymmetric bead attachments. The results, presented in Fig 9 also demonstrate the existence of an anomalous propulsion regimes as highlighted by the bands in cyan color in the three graphs on the right. The experimental value of 〈Ω_*z*_〉/*ω*_0_ ∼ 0.004 (total rotation of ∼ *π*/4 in 1299 msec; see S7 Video) is slightly larger than the values 0.0024, 0.0029 and 0.0028 in panels A-C, respectively, which are highlighted by the red circles in Fig 9A–9C. Finally, comparing Figs 8 and 9 shows that, as expected, the dependence of the mean translational and rotational velocities on the bead size is highly sensitive to the flagellar waveform.

## 4 Conclusions

In this work, we have studied analytically and by numerical simulations the motion of a bead propelled by a model flagellum. We used data from our previous experimental study in which isolated and demembranated flagella of the green alga *C. reinhardtii* were reactivated with ATP to propel a bead. [14]. In this work, we observed two distinct regimes of bead propulsion depending on the calcium concentration. The first regime describes the bead motion along a curved trajectory which is observed in experiments at zero or very small concentration of calcium ions (less than 0.02 mM). In the second regime and at higher calcium concentrations, the cargo is propelled along a straight trajectory, at an averaged velocity as high as ∼20 *μ*m/sec, comparable to the typical human sperm migration speed in mucus [36]. Calcium ions are known to affect the flagellar waveform by reducing the mean curvature (*C*_0_) of axonemes in a dose-dependent manner [22, 23], thereby inducing a transition from circular to straight swimming trajectories.

To characterize the motion, we first used a simplified waveform to describe the axonemal shapes which is composed of a traveling wave component propagating along a circular arc (Eq 19). This simplified waveform allows us to obtain analytical expressions for the translational and rotational velocities of an axonemally-propelled bead in the limit of small amplitudes of curvature waves. The rotational velocity of an axoneme is predominately controlled by its mean curvature *C*_0_. As shown in Ref. [19], the second harmonics (as well as higher harmonics of even order) of the flagellar waveform also contribute to the rotational velocity of an axoneme, although more weakly (at higher orders). Remarkably, our analysis with the simplified waveform predicts a non-monotonous dependence of the rotational velocity, and/or of some of the components of the translational velocity as a function of the size of the bead. Namely, some of these components may increase when the size of the bead, hence the overall drag, increases, see Fig 5. It is also very interesting to note that the translational velocity components *U*_*x*_ and *U*_*y*_ are nearly saturated for a fairly large range of cargo size.

Further, we used our experimental beat patterns from Ref. [14] to demonstrate that this counter-intuitive regime is not limited to the simplified waveform and also exists for waveforms closer to the experimental ones. This anomalous propulsion regime has also been predicted for a model sperm-like swimmer with a zero mean curvature, propelled by a traveling wave component. Consistent with this, we also observed anomalous regimes using our experimental beat patterns from Ref. [14] at an increased calcium concentration (0.1 mM instead of 0 mM), in which the mean curvature of axonemes is significantly reduced (by a factor of about 10).

An anomalous cargo transport regime was also predicted in biofilm forming bacteria *Pseudomonas aeruginosa* (PA14) [12, 13], where swimming is driven by multiple (on average two) rotating helical flagella (length∼4 *μ*m) that can bundle to propel the bacterium in a corkscrew-like motion or unbundle to change direction, exhibiting a run-and-tumble swimming pattern [37]. This anomalous behavior is expected to exist for a hypothetical mutant of PA14 which has a larger (around three times) size than the wild type. This up-scaling of bacteria size results in a larger rotational drag coefficient of the flagellum, compared to that of the bacterial body, which in Ref. [12] appears to be as criterion for anomalous propulsion. Whether such a hypothetical large-scale bacterium exists is an open question. However, this anomalous propulsion regime could be important in bacterial swimming in polymeric solutions, where due to steric interactions between flagella and polymers, the rotational drag coefficient of the flagella can become larger than that of the bacterial body, fulfilling the criteria for anomalous propulsion. Thus, as experimentally observed and contrary to our expectations, the swimming speed of bacteria in the polymeric solutions can increase [38, 39]. In our system, however, we are not able to obtain a simple analytical criteria for the anomalous propulsion as it results from the full calculations which include inverting the full time-dependent, 3 × 3 drag matrix, *D*_*A*_ + *D*_*B*_, see Eqs 16 and 17 of the bead-axoneme swimmer. From a general physics perspective, we remark that the anomalous regime corresponds to a change in the partitioning between translation (in the two physical directions) and rotation as *R* increases, so that some components may increase with *R* over a range while other components decrease or remain almost constant over a fairly large range of the cargo size, as imposed by the overall increase of the drag due to the bead.

Furthermore, our analysis shows that asymmetric cargo-axoneme attachment provides a contribution to the rotational velocity, comparable to that of the mean curvature of the flagellum. In other words, a sperm-like beating flagellum without mean curvature and second harmonic swims in a curved trajectory if it is attached sideways to a cargo. In our experiments [14], the limitations due to the 2D imaging technique prevented us from precisely distinguishing symmetric versus asymmetric bead-axoneme attachments. Indeed, in a 2D-projected image, a symmetric bead-axoneme attachment could in reality be an asymmetric one. Moreover, as the bead-axoneme swimmer goes slightly out of focus, the attachment in some frames seems to be symmetric and in other frames asymmetric. The 3D microscopy techniques utilized in Ref. [25] are necessary to distinguish a symmetric from an asymmetric axoneme-bead attachment. This 3D characterization is absolutely essential to experimentally prove the anomalous behavior predicted by our analysis with a simplified waveform as well as with the experimental beat patterns. Although in Ref. [14], we performed few experiments with beads of diameters of 1, 2 and 3 *μ*m, we are unable to verify the validity of the predicted anomalous trend because the 2D microscopy does not allow us to distinguish between symmetric and asymmetric bead-axoneme attachment.

Finally, it is important to note that in our analysis we have assumed that the presence of the bead (load) does not affect the waveform of the flagellum. S4 Fig in S1 File shows the hydrodynamic force distribution along the contour length of a flagellum with a given waveform at a fixed time and for different bead radii, and indeed we see that the force distribution depends on the size of the bead. These tangential and perpendicular components of the hydrodynamic force (which vary with the bead size) might feedback into the activity of the dynein molecular motors and thereby, change the flagellar waveform. By taking the product of the force distribution with the velocity distribution and integrating, we find the hydrodynamic energy dissipation to range between 0.01 × ∼ 10^−15^ J/s (*r* = 0) and 0.17 × 10^−15^ J/s (*r* = 0.5). From the energy budget point of view, the ATP consumption measurements at the single-axoneme level [15] show that the energy required to generate elastic deformation in an axoneme is one order of magnitude larger than hydrodynamic dissipation. The WT active *Chlamydomonas* axonemes consume approximately 10^6^ ATP molecules/s, corresponding to an energy consumption of 8.1 × 10^−14^ J/s [15]. It is estimated that this energy is expended primarily for elastic deformation of the flagellum and not for overcoming viscous drag, as they calculate the viscous losses as 6.4 × 10^−15^ J/s [15]. This suggests that the feedback effect of hydrodynamic drag on motor activity might be negligible. In-depth studies to incorporate the feedback between motor activity and hydrodynamic forces require a microscopic description of flagellar dynamics and are the subject of our future work.

The design and fabrication of synthetic micro-swimmers is a challenging task in the growing field of smart drug delivery, and has recently become a multidisciplinary effort involving physicists, biologists, chemists and materials scientists. Our theoretical analysis as well as numerical simulations reveal the existence of an anomalous cargo transport regime, where contrary to expectation, the flagellar-propelled cargo rotates faster as we increase the cargo size. This counter-intuitive behavior may play a crucial role in the design of future artificial flagellar-based propulsion systems, where targeted transport of cargo is the goal and higher rotational speeds could reduce the efficiency of directional propulsion. Finally, our analysis also highlights the contribution of the asymmetric cargo-flagellum attachment in the rotational velocity of the micro-swimmer. This turning mechanism should be also taken into account in manufacturing bio-inspired synthetic swimmers where a directional targeted motion is critical for delivery of drug-loaded cargoes.

## Supporting information

S1 File Supplementary material to the manuscript. (PDF)

S1 Video Experiment: An axonemally-driven bead. An isolated and demembranated flagellum from green algae C. reinhardtii is attached to a 1 micron-sized bead. The axoneme is reactivated with 1 mM ATP and beats at around 110 Hz.
(MOV)

S2 Video Experiment: Bead trajectory. Trajectory of the bead in S1 Video. Over time, the bead is propelled on a helical-like trajectory (blue curve).
(MOV)

S3 Video Simulations: Symmetric versus asymmetric bead-axoneme attachment. The simulation of a bead symmetrically attached to a flagellum shows that it swims on a straight path. The flagellar beat pattern consists only of the traveling wave component *C*_1_ (*C*_0_ = 0); see also Fig 7A.
(MOV)

S4 Video Simulations: Symmetric versus asymmetric bead-axoneme attachment. The simulation of a bead asymmetrically attached to a flagellum shows that it swims on a curved path. The flagellar beat pattern consists only of the traveling wave component *C*_1_ (*C*_0_ = 0); see also Fig 7B.
(MOV)

S5 Video Simulations: Symmetric versus asymmetric bead-axoneme attachment. The simulation of a bead asymmetrically attached to a flagellum shows that it swims on a curved path. The flagellar beat pattern consists only of the traveling wave component *C*_1_ (*C*_0_ = 0); see also Fig 7C.
(MOV)

S6 Video Experiment without calcium. Experimental beat pattern reported in Ref. [14] used for the analysis in Fig 8. Here [Ca^2+^] = 0 mM and [ATP] = 80 *μ*M.
(MOV)

S7 Video Experiment with calcium. Experimental beat pattern reported in Ref. [14] with [Ca^2+^] = 0.1 mM and [ATP] = 80 *μ*M.
(MOV)
