# Supplementary material for: Flagellum-driven cargoes: Influence of cargo size and the flagellum-cargo attachment geometry
Source: PLoS One. 2023 Mar 10;18(3):e0279940. doi: 10.1371/journal.pone.0279940 (PMC10004597; doi:10.1371/journal.pone.0279940)
Supplement: S1 File — (PDF) [file pone.0279940.s001.pdf]

Supporting Information to: Flagellum-driven cargoes: influence of cargo size and the flagellum-cargo attachment geometry

Albert J Bae[1], Raheel Ahmad[2,3], Eberhard Bodenschatz[2,4,5], Alain Pumir[2,6], Azam Gholami[2,7*]

1 Lewis & Clark College, Portland, Oregon, USA
2 Max Planck Institute for Dynamics and Self-Organization, Am Fassberg 17, D-37077 Göttingen, Germany
3 Center for Cancer Research and Center for Engineering in Medicine and Surgery, Harvard Medical School and Massachusetts General Hospital, Charlestown, 02129 Massachusetts, USA
4 Institute for Dynamics of Complex Systems, University of Göttingen, Göttingen 37077, Germany
5 Laboratory of Atomic and Solid-State Physics and Sibley School of Mechanical and Aerospace Engineering, Cornell University, Ithaca, New York 14853, USA
6 Laboratoire de Physique, Ecole Normale Supérieure de Lyon, Université Lyon 1 and CNRS, F-69007 Lyon, France
7 New York University Abu Dhabi, Abu Dhabi, United Arab Emirates

* azam.gholami@nyu.edu

1 Drag matrix of a bead in 3D

Although our analysis in the current work is limited to 2D, we also present the drag matrix of the bead in 3D with respect to the coordinate system which is defined to be the bead-axoneme attachment point. Extending the full analysis to 3D is the subject of our future work.

Let us fix a couple of points P and P' on or in a rigid body (see Fig. S1A). The distance between these points remains constant. Furthermore, if we attach two parallel vectors at P and P', they would remain parallel under movement. Since this is the case, it follows that $\mathbf{\Omega}_P = \mathbf{\Omega}_{P'}$, so we will drop the subscript and call it $\mathbf{\Omega}$.

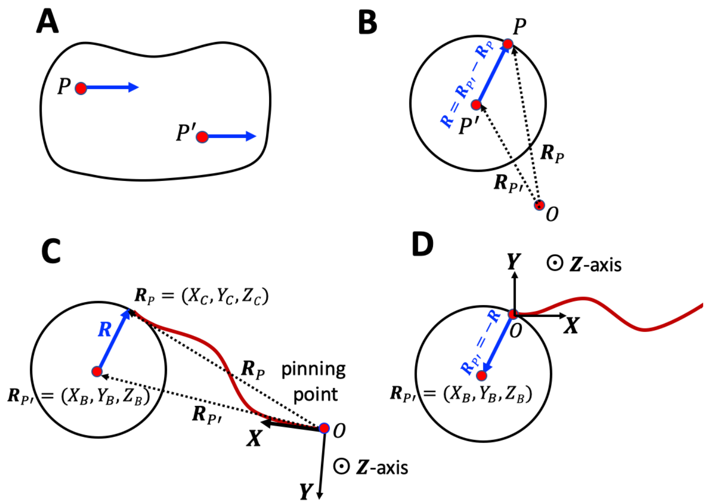

Fig S1. Schematic presentation of the set up and the coordinate system.

Let us denote the positions of P and P' by $\mathbf{R}_P$ and $\mathbf{R}_{P'}$ (Fig. S1B). The distance $|\mathbf{R}_{P'} - \mathbf{R}_P|$ is constant, but the orientation changes:

$$\mathbf{U}_{P'} - \mathbf{U}_P = \frac{d}{dt}(\mathbf{R}_{P'} - \mathbf{R}_P) = -(\mathbf{R}_{P'} - \mathbf{R}_P) \times \boldsymbol{\Omega}. \tag{S.1}$$

Next, suppose we apply a distribution of forces $\mathbf{f}_i$ at $\mathbf{R}_i$ as $\mathbf{F} = \Sigma_i \mathbf{f}_i$ which does not depend on P and P'. The torques depend on P and P':

$$\boldsymbol{\tau}_P = \Sigma_i(\mathbf{R}_i - \mathbf{R}_P) \times \mathbf{f}_i, \quad \text{and} \quad \boldsymbol{\tau}_{P'} = \Sigma_i(\mathbf{R}_i - \mathbf{R}_{P'}) \times \mathbf{f}_i, \tag{S.2}$$

and the difference gives:

$$\boldsymbol{\tau}_{P'} - \boldsymbol{\tau}_P = -(\mathbf{R}_{P'} - \mathbf{R}_P) \times \mathbf{F}. \tag{S.3}$$

Now let us define the problem we wish to solve: What is the force $\mathbf{F} = (F_x, F_y, F_z)^T$ and torque $\boldsymbol{\tau} = (\tau_x, \tau_y, \tau_z)^T$ we need to apply at point P on the bead's surface to make the bead move with translational velocity $\mathbf{U} = \mathbf{U}_P$ and angular velocity $\boldsymbol{\Omega}$? This amounts to asking what is the drag force and drag torque one needs to overcome. Let us denote the center of the bead as P' and define $\mathbf{R} = \mathbf{R}_P - \mathbf{R}_{P'}$ (Fig. S1B). This problem would have been much simpler if we were asked what force $\mathbf{F}'$ and torque $\boldsymbol{\tau}'$ needs to be applied at the bead's center to counteract drag:

$$\begin{pmatrix} \mathbf{F}' \\ \boldsymbol{\tau}' \end{pmatrix} = \begin{pmatrix} \nu_T \mathbf{I} & \mathbf{O} \\ \mathbf{O} & \nu_R \mathbf{I} \end{pmatrix} \begin{pmatrix} \mathbf{U}' \\ \boldsymbol{\Omega}' \end{pmatrix}. \tag{S.4}$$

Here $\nu_T = 6\pi R\mu$, $\nu_R = 8\pi R^3 \mu/3$ and:

$$\mathbf{I} = \begin{pmatrix} 1 & 0 & 0 \\ 0 & 1 & 0 \\ 0 & 0 & 1 \end{pmatrix}, \quad \text{and} \quad \mathbf{O} = \begin{pmatrix} 0 & 0 & 0 \\ 0 & 0 & 0 \\ 0 & 0 & 0 \end{pmatrix}. \tag{S.5}$$

Now we use what we learned about rigid body motion in Eq. S.1:

$$\begin{pmatrix} \mathbf{U}' \\ \boldsymbol{\Omega}' \end{pmatrix} = \begin{pmatrix} \mathbf{U} - (\mathbf{R}_{P'} - \mathbf{R}_P) \times \boldsymbol{\Omega} \\ \boldsymbol{\Omega} \end{pmatrix} = \begin{pmatrix} \mathbf{U} + \mathbf{R} \times \boldsymbol{\Omega} \\ \boldsymbol{\Omega} \end{pmatrix} = \begin{pmatrix} \mathbf{I} & \boldsymbol{\mathcal{R}} \\ \mathbf{O} & \mathbf{I} \end{pmatrix} \begin{pmatrix} \mathbf{U} \\ \boldsymbol{\Omega} \end{pmatrix}, \tag{S.6}$$

where we have defined:

$$\boldsymbol{\mathcal{R}} = \begin{pmatrix} 0 & R_z & R_y \\ R_z & 0 & -R_x \\ -R_y & R_x & 0 \end{pmatrix} = \begin{pmatrix} 0 & -Z_C + Z_B & Y_C - Y_B \\ Z_C - Z_B & 0 & -X_C + X_B \\ -Y_C + Y_B & X_C - X_B & 0 \end{pmatrix}. \tag{S.7}$$

Recall that $\mathbf{R}_P = (X_C, Y_C, Z_C)$ are the coordinates of the flagella-bead contact point and $\mathbf{R}_{P'} = (X_B, Y_B, Z_B)$ are the coordinates of the bead center (Fig. S1C). Similarly, we have that:

$$\begin{pmatrix} \mathbf{F}' \\ \boldsymbol{\tau}' \end{pmatrix} = \begin{pmatrix} \mathbf{F} \\ \boldsymbol{\tau} - (\mathbf{R}_{P'} - \mathbf{R}_P) \times \mathbf{F} \end{pmatrix} = \begin{pmatrix} \mathbf{F} \\ \boldsymbol{\tau} + \mathbf{R} \times \mathbf{F} \end{pmatrix} = \begin{pmatrix} \mathbf{I} & \mathbf{O} \\ \boldsymbol{\mathcal{R}} & \mathbf{I} \end{pmatrix} \begin{pmatrix} \mathbf{F} \\ \boldsymbol{\tau} \end{pmatrix}, \tag{S.8}$$

so the Eq. S.4 becomes:

$$\begin{pmatrix} \mathbf{I} & \mathbf{O} \\ \boldsymbol{\mathcal{R}} & \mathbf{I} \end{pmatrix} \begin{pmatrix} \mathbf{F} \\ \boldsymbol{\tau} \end{pmatrix} = \begin{pmatrix} \nu_T \mathbf{I} & \mathbf{O} \\ \mathbf{O} & \nu_R \mathbf{I} \end{pmatrix} \begin{pmatrix} \mathbf{I} & \boldsymbol{\mathcal{R}} \\ \mathbf{O} & \mathbf{I} \end{pmatrix} \begin{pmatrix} \mathbf{U} \\ \boldsymbol{\Omega} \end{pmatrix}. \tag{S.9}$$

Multiplying both sides by $\begin{pmatrix} \mathbf{I} & \mathbf{O} \\ -\boldsymbol{\mathcal{R}} & \mathbf{I} \end{pmatrix}$ and calculating the products of matrices yields:

$$\begin{pmatrix} \mathbf{F} \\ \boldsymbol{\tau} \end{pmatrix} = \begin{pmatrix} \nu_T \mathbf{I} & \nu_T \boldsymbol{\mathcal{R}} \\ -\nu_T \boldsymbol{\mathcal{R}} & \nu_R \mathbf{I} - \nu_T \boldsymbol{\mathcal{R}}^2 \end{pmatrix} \begin{pmatrix} \mathbf{U} \\ \boldsymbol{\Omega} \end{pmatrix}, \tag{S.10}$$

where

$$\mathcal{R}^2 = \begin{pmatrix} 0 & -R_z & R_y \\ R_z & 0 & -R_x \\ -R_y & R_x & 0 \end{pmatrix} \begin{pmatrix} 0 & -R_z & R_y \\ R_z & 0 & -R_x \\ -R_y & R_x & 0 \end{pmatrix} \tag{S.11}$$

$$= \begin{pmatrix} -R_y^2 - R_z^2 & R_xR_y & R_xR_z \\ R_xR_y & -R_x^2 - R_z^2 & R_yR_z \\ R_xR_z & R_yR_z & -R_x^2 - R_y^2 \end{pmatrix}. \tag{S.12}$$

Possibly noteworthy is that:

$$\mathcal{R}^2 + R^2\mathbf{I} = \begin{pmatrix} -R_y^2 - R_z^2 & R_xR_y & R_xR_z \\ R_xR_y & -R_x^2 - R_z^2 & R_yR_z \\ R_xR_z & R_yR_z & -R_x^2 - R_y^2 \end{pmatrix} + \begin{pmatrix} R^2 & 0 & 0 \\ 0 & R^2 & 0 \\ 0 & 0 & R^2 \end{pmatrix}$$

$$= \begin{pmatrix} R_x^2 & R_xR_y & R_xR_z \\ R_xR_y & R_y^2 & R_yR_z \\ R_xR_z & R_yR_z & R_z^2 \end{pmatrix} = \mathbf{R}\mathbf{R}^T. \tag{S.13}$$

For the motion in $x-y$ plane (2D), we are only interested in F_x, F_y and τ_z as a function of U_x, U_y and Ω_z. Here we also assume $R_z = 0$, so we are only interested in components 1, 2, and 6:

$$\begin{pmatrix} F_x \\ F_y \\ \tau_z \end{pmatrix} = \begin{pmatrix} \nu_T & 0 & \nu_T\mathcal{R}_{13} \\ 0 & \nu_T & \nu_T\mathcal{R}_{23} \\ -\nu_T\mathcal{R}_{31} & -\nu_T\mathcal{R}_{32} & \nu_R - \nu_T\mathcal{R}_{33}^2 \end{pmatrix} \begin{pmatrix} U_x \\ U_y \\ \Omega_z \end{pmatrix}$$

$$= \begin{pmatrix} \nu_T & 0 & \nu_T R_y \\ 0 & \nu_T & -\nu_T R_x \\ \nu_T R_y & -\nu_T R_x & \nu_R + R^2\nu_T \end{pmatrix} \begin{pmatrix} U_x \\ U_y \\ \Omega_z \end{pmatrix}. \tag{S.14}$$

Up until now, we haven't actually specified where our origin is. Let us set the origin to be at point P on the surface of the bead ($\mathbf{R}_P = 0$). We will choose $\mathbf{X}$ to point tangent to the flagella at the point of attachment, and let the coordinates of the center of the bead be located at $\mathbf{R}_{P'} = (X_B, Y_B, 0)$. Note that $X_B \leq 0$ and $-R \leq Y_B \leq R$ (Fig. S1D). Also note that $\mathbf{R} = -\mathbf{R}_{P'}$, so the force equation reads:

$$\begin{pmatrix} F_x \\ F_y \\ \tau_z \end{pmatrix} = \begin{pmatrix} \nu_T & 0 & -\nu_T Y_B \\ 0 & \nu_T & \nu_T X_B \\ -\nu_T Y_B & \nu_T X_B & \nu_R + \nu_T R^2 \end{pmatrix} \begin{pmatrix} U_x \\ U_y \\ \Omega_z \end{pmatrix}, \tag{S.15}$$

which was previously extracted in Eq. 14. Note that setting $Y_B \neq 0$ allows us to handle the case where the flagella is not attached normal to the bead.

2 Rotational and translational velocities of an axoneme attached symmetrically to a bead

We used the simplified waveform given by Eq. 19 to calculate translational and rotational velocities of a freely swimming axoneme attached symmetrically from the basal end to a bead of dimensionless radius $r = R/L$. In the limit of small $C_0 = \kappa_0 L/(2\pi)$ and $C_1 = \kappa_1 L/(2\pi)$, we calculate rotational and translational velocities of the swimmer from Eq. 4 by calculating the propulsive forces and inverting the drag matrix $\mathbf{D}$ (see Materials and Methods). We then take average over one beat cycle to obtain:

$$\frac{\langle \Omega_z \rangle}{\omega_0} \approx \frac{7C_0C_1^2\zeta'_\perp}{5\pi^3\left(7\eta\zeta'_\perp + 96\pi r\right)\left(73728\pi^2 r^4 + 2688\zeta'_\perp\pi(r(5r+3)+1)r + 49\zeta'^2_\perp\right)^2}\big(5\big(24461180928$$

$$\times(\eta-1)\pi^8 r^7+42467328\pi^6(-720\eta+32(9\eta-11)\pi^2+7(\eta-1)(2\eta+9)\zeta'_\perp\pi+240)r^6$$
$$+3096576\zeta'_\perp\pi^5(-24\eta(26\eta+67)+16(\eta(8\eta+31)-47)\pi^2+35(\eta-1)\zeta'_\perp\pi+552)r^5$$
$$+258048\zeta'_\perp\pi^4(288\pi(4\pi^2\eta-21\eta-4\pi^2+9)+7\zeta'_\perp(-3\eta(65\eta+23)+(\eta(8\eta+67)$$
$$-83)\pi^2+24))r^4+2688\zeta'_\perp\pi^3(-735(\eta(9\eta-4)+1)\zeta'^2_\perp+14112(\eta-1)\pi^3\zeta'_\perp-6048$$
$$\times(\eta+3)(3\eta-1)\pi\zeta'_\perp+18432(\eta-1)\pi^4+(245(2\eta^2+\eta-3)\zeta'^2_\perp-27648(5\eta-3))\pi^2$$
$$+82944(2\eta-1))r^3+56448\zeta'^2_\perp\pi^2(112\pi^4(\eta-1)+7(\eta+2)\zeta'_\perp\pi^3(\eta-1)-24(\eta(4\eta+31)$$
$$-17)\pi^2+288(\eta+2)(2\eta-1)+21\eta(1-5\eta)\zeta'_\perp\pi)r^2+32928\zeta'^3_\perp\pi(7\pi^4(\eta-1)-3(\eta(13\eta$$
$$+5)-6)\pi^2+36(4\eta^2-1))r-2401\zeta'^4_\perp(9(-8+\pi^2)\eta^2-(-36+\pi^4)\eta+\pi^4-3\pi^2)\Big)\Big), \quad \text{(S.16)}$$

$$\frac{\langle U_x\rangle}{L\omega_0}\approx-\frac{21C_1^2\zeta'_\perp}{6\pi^2\left(49\zeta'^2_\perp+2688\pi\zeta'_\perp(r(5r+3)+1)r+73728\pi^2r^4\right)\left(7\eta\zeta'_\perp+96\pi r\right)}\left(49(6\eta+\pi^2-3)\zeta'^2_\perp\right.$$
$$\left.-672\pi\zeta'_\perp r(2\eta(\pi^2(r(20r+9)+2)-3)-\pi^2(2r(20r+9)+5)+3)-221184\pi^4(\eta-1)r^4\right), \quad \text{(S.17)}$$

$$\frac{\langle U_y\rangle}{L\omega_0}\approx-\frac{7C_0C_1^2\zeta'_\perp}{30\pi^3\left(7\eta\zeta'_\perp+96\pi r\right)\left(73728\pi^2r^4+2688\zeta'_\perp\pi\left(r(5r+3)+1\right)r+49\zeta'^2_\perp\right)^2}\Big(45$$
$$\times\Big(7\zeta'_\perp(-4718592\pi^5(4\pi^2(5r+2)-39)r^6+516096\zeta'_\perp\pi^2(\pi^2(r(35r-6)+1)-6)r^3$$
$$+3136\zeta'^2_\perp\pi(4\pi^4(5r+2)r-72r-3\pi^2(r(90r+29)+6)+36)r-1029\zeta'^3_\perp(-8+\pi^2))\eta^2$$
$$+(-1358954496\pi^6(\pi^2(20r+6)-15)r^7-16515072\zeta'_\perp\pi^3(-3\pi^2(33r^2+6r+5)$$
$$+2\pi^4(r(r(70r+39)+12)+1)+18)r^4-301056\zeta'^2_\perp\pi^2(36(r-1)+3\pi^2(r(80r^2+74r+39)$$
$$+10)+2\pi^4(r(2r(5r(20r+19)+46)+25)+4))r^2-10976\zeta'^3_\perp\pi(-36(2r+1)$$
$$+2\pi^4(4r+1)(5r+2)-3\pi^2(4r(25r+6)+1))r+2401\zeta'^4_\perp(-12+\pi^2))\eta+32\pi r(14155776\pi^5$$
$$\times(\pi^2(60r+22)-15)r^6+516096\zeta'_\perp\pi^2(-3\pi^2(r(13r+6)+3)+2\pi^4(r(r(90r+49)+9)+1)+9)$$
$$\times r^3+9408\zeta'^2_\perp\pi(36r+3\pi^2(20r^3+2r^2+r+2)+2\pi^4(r(2r(5r(20r+17)+31)+15)+2)-18)r$$
$$+343\zeta'^3_\perp(\pi^4(6r+2)-3\pi^2(4r+1)(10r+1)-36))\Big)\Big). \quad \text{(S.18)}$$

Here $\zeta'_\perp=\zeta_\perp/\mu=2\zeta_\parallel/\mu\sim 8\pi/(\log(2L/a)+0.5)\sim 4.33$ where $a\sim 0.1$ μm is the radius of axoneme, $L\sim 10$ μm is the contour length of axoneme and we have assumed $\eta=\zeta_\parallel/\zeta_\perp=0.5$. Exemplary, for $r=R/L=0.1$ and with $\eta=0.5$, Eqs. S.16-S.18 simplify to:

$$\frac{\langle\Omega_z\rangle}{\omega_0}\approx-0.24C_0C_1^2, \quad \text{(S.19)}$$
$$\frac{\langle U_x\rangle}{L\omega_0}\approx-0.04C_1^2, \quad \text{(S.20)}$$
$$\frac{\langle U_y\rangle}{L\omega_0}\approx+0.006C_0C_1^2. \quad \text{(S.21)}$$

3 Rotational velocity of a bead attached asymmetrically to a freely-swimming axoneme

To illustrate the analytical results, we consider the special case of a model axoneme where only the main traveling wave component C_1 is present, and set the static component C_0 to zero. A bead is attached asymmetrically to a flagellum at positions X_B and Y_B, as shown schematically in Fig. 3A. We calculate the mean rotational velocity of the swimmer using the matrix introduced

in Eq. S.15 and drag matrix of the flagellum to obtain:

$$\langle\Omega_z\rangle/\omega_0 \approx -\Bigg(8064C_1^2\zeta'_\perp r y_b\Big(7\eta^2\zeta'_\perp(343\zeta'^3_\perp(6+\ \pi^2)-2352\zeta'^2_\perp\pi r(-24+\pi^2 \times(-3+40r^2-12x_b))-5308416\pi^5r^3(5r^2-3x_b^2)-129024\zeta'_\perp\pi^2r^2 \times(-3+\pi^2(-1+25r^2-12x_b^2)))+288\pi r(-343\zeta'^3_\perp+21504\zeta'_\perp\pi^2r^2 \times(-3+\pi^2(-1+50r^2-24x_b^2-30y_b^2))+784\zeta'^2_\perp\pi r(-12+\pi^2(1+40r^2 -12x_b-24y_b^2))+3538944\pi^5r^5)+3\eta\big(-2401\zeta'^4_\perp+5488\zeta'^3_\perp\pi^3r \times(3+40r^2-12x_b)-4128768\zeta'_\perp\pi^3r^3(-3+\pi^2(-1+10r^2-3x_b^2-15y_b^2)) -339738624\pi^6r^6+75264\zeta'^2_\perp\pi^2r^2\times(18+\pi^2(1+60r^2+12(1-4x_b)x_b +24y_b^2)))\Big)\Bigg)\Bigg/\Bigg(2\pi^2\Big((7\eta\zeta'_\perp+96\pi r)(49\zeta'^2_\perp+2688\zeta'_\perp\pi r(1+5r^2-3x_b) +36864\pi^2r^2(5r^2-3x_b^2))-110592\pi^2r^2(7\zeta'_\perp+96\pi r)y_b^2\Big)^2\Bigg), \tag{S.22}$$

where $x_b = X_B/L$, $y_b = Y_B/L$, $r = R/L$, $x_b^2+y_b^2 = r^2$, $\zeta'_\perp = \zeta_\perp/\mu = 4.33$ and $\eta = \zeta_\parallel/\zeta_\perp = 0.5$.

Note that the expressions for $\langle U_x\rangle$ and $\langle U_y\rangle$ are excessively long, and not worth presenting here. In the following we show the results for the restrictive case, shown in Fig. 7B where $x_b = 0$ and $y_b = -r$, and C_0 is set to zero:

$$\langle U_x\rangle/L\omega_0 \approx \Bigg(7C_1^2\zeta'_\perp(-16807\eta(6\eta+\pi^2-3)\zeta'^5_\perp+230496\pi(\eta((4\pi^2-30)\eta-9\pi^2+9)-\pi^2+3)\zeta'^4_\perp r +37933056\pi^2(-2\eta(10\eta+3)+\pi^2(\eta(25\eta-29)-2)+8)\zeta'^3_\perp r^4+3161088\pi^2(16\pi^2(\eta-1)\eta -6(4\eta+3)\eta-9\pi^2+15)\zeta'^3_\perp r^2+3468165120\pi^5(\eta-1)(25\eta+4)\zeta'^2_\perp r^7+2064384\pi^4\zeta'_\perp r^6 (1225(\eta-1)\eta\zeta'^2_\perp+4608(5\pi^2-12)\eta-4608(7\pi^2-6))+173408256\pi^3(\pi^2(16\eta(5\eta-4) -37)-3(2\eta-1)(5\eta+16))\zeta'^2_\perp r^5+18816\pi\zeta'^2_\perp r^3(735\eta(1-2\eta)\zeta'^2_\perp+\pi^2(245\eta(2\eta-3)\zeta'^2_\perp -27648(2\eta-1))+9216\pi^4(4\eta-5))+237817036800\pi^6(\eta-1)(3\eta+2)\zeta'_\perp r^8 +3913788948480\pi^7(\eta-1)r^9)\Bigg)\Bigg/\Bigg(4\pi^3(7\eta\zeta'_\perp(49\zeta'^2_\perp+2688\pi\zeta'_\perp(5r^2+1)r+184320\pi^2r^4) +96\pi r(49\zeta'^2_\perp+2688\pi\zeta'_\perp(2r^2+1)r+73728\pi^2r^4))^2\Bigg), \tag{S.23}$$

$$\langle U_y\rangle/L\omega_0 \approx \Bigg(14112C_1^2\zeta'^2_\perp r^2(-343\eta((6+\pi^2)\eta-3)\zeta'^3_\perp+94080\pi^3(\eta-1)\eta\zeta'^2_\perp r^3-4704\pi(\eta(6\eta+2\pi^2+3) -3)\zeta'^2_\perp r+516096\pi^4(\eta-1)(5\eta+1)\zeta'_\perp r^4-64512\pi^2(6\eta+\pi^2-3)\zeta'_\perp r^2+14155776\pi^5(\eta-1) \times r^5)\Bigg)\Bigg/\Bigg(\pi^2(7\eta\zeta'_\perp(49\zeta'^2_\perp+2688\pi\zeta'_\perp(5r^2+1)r+184320\pi^2r^4)+96\pi r(49\zeta'^2_\perp+2688\pi\zeta'_\perp (2r^2+1)r+73728\pi^2r^4))^2\Bigg). \tag{S.24}$$

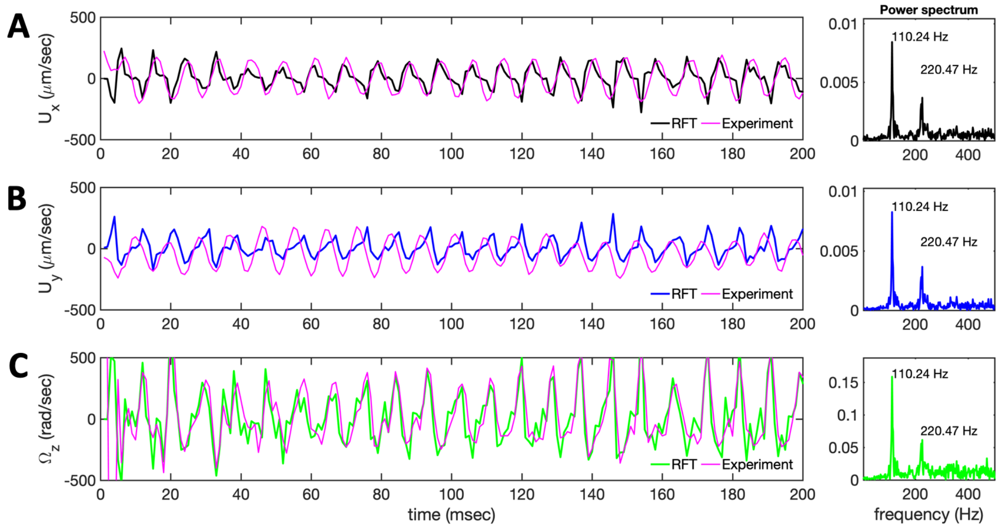

Fig S2. A-C) Velocity components of the bead's center $U_x(t)$ and $U_y(t)$, and the rotational velocity of the bead $\Omega_z(t)$ measured in the body-fixed frame of the exemplary axoneme in Fig. 1. Comparison with results obtained in the framework of RFT shows a good, semi-quantitative agreement, in particular for the rotational velocity, $\Omega_z(t)$ (panel C). The power spectra show a dominant peak at the beat frequency of 110.24 Hz and its second harmonic. $[\text{ATP}] = 1$ mM and $[\text{Ca}^{2+}] = 0$ mM.

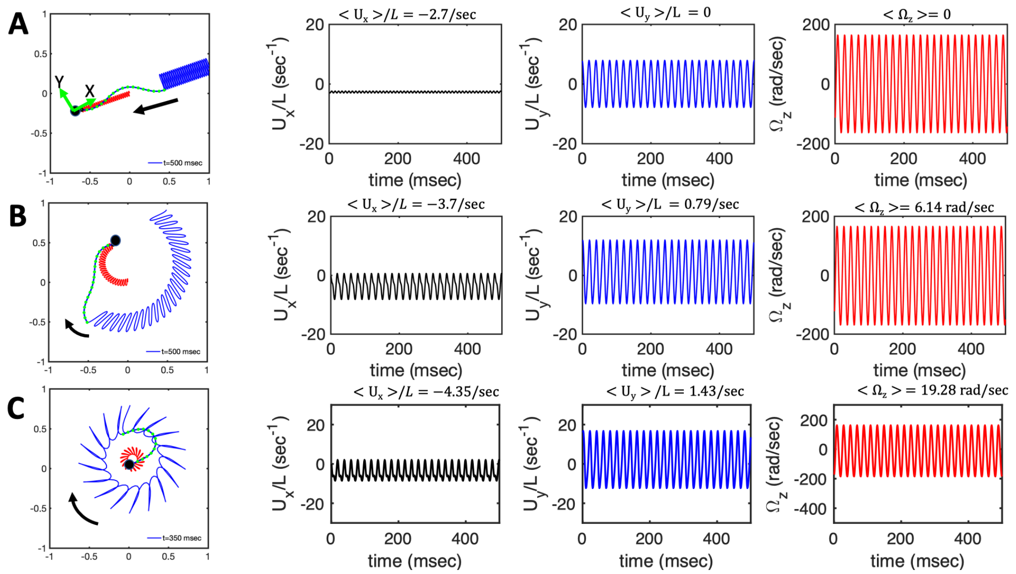

Fig S3. Simulations to show the effect of C_0 at a fixed amplitude of dynamic mode $C_1 = 0.5$. A bead of radius $R/L = 0.1$ is attached to the basal end. A-C) The swimming trajectory and mean translational and rotational velocities of the swimmer in the body-fixed frame. Parameters are $f_0 = 50$ Hz, $\eta = \zeta_{||}/\zeta_{\perp} = 0.5$, A) $C_0 = 0$, B) $C_0 = 0.25$, and C) $C_0 = 0.5$.

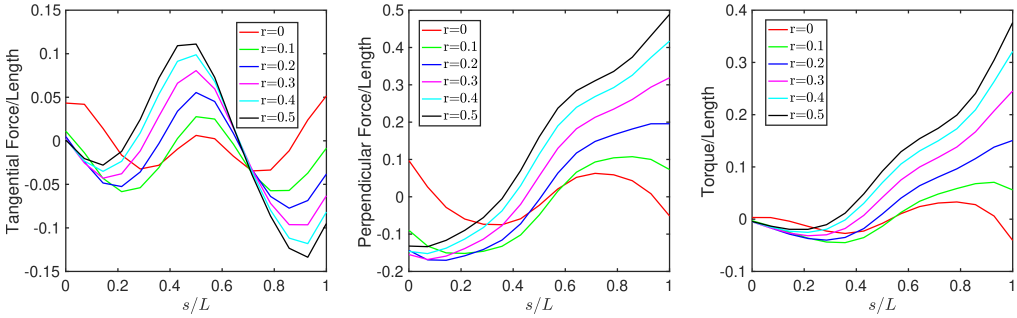

Fig S4. The dimensionless distribution of the hydrodynamic force and torque per unit length, measured as the sum of the driving force and the drag force along the flagellum length for a given flagellar waveform ($C_0 = 0$, $C_1 = 0.9$, $f = 50$ Hz and $\lambda = L$ in Eq. 19) at a fixed time ($t = 50$ msec), for different bead radii $r = R/L$.
